# HIV-1 infection of genetically engineered iPSC-derived central nervous system-engrafted microglia in a humanized mouse model

**DOI:** 10.1128/jvi.01595-23

**Published:** 2023-11-30

**Authors:** Alice K. Min, Behnam Javidfar, Roy Missall, Donald Doanman, Madel Durens, Mara Graziani, Annika Mordelt, Samuele G. Marro, Lotje de Witte, Benjamin K. Chen, Talia H. Swartz, Schahram Akbarian

**Affiliations:** 1 Division of Infectious Diseases, Department of Medicine, Icahn School of Medicine at Mount Sinai, New York, New York, USA; 2 Nash Family Department of Neuroscience, Friedman Brain Institute, Icahn School of Medicine at Mount Sinai, New York, New York, USA; 3 Department of Psychiatry, Icahn School of Medicine at Mount Sinai, New York, New York, USA; 4 Black Family Stem Cell Institute, Icahn School of Medicine at Mount Sinai, New York, New York, USA; 5 Department of Human Genetics and Department of Cognitive Neuroscience, Radboud UMC, Nijmegen, the Netherlands; 6 Centre for Neuroscience, Donders Institute for Brain, Cognition, and Behavior, Nijmegen, the Netherlands; Ulm University Medical Center, Ulm, Germany

**Keywords:** HIV-1, microglia, induced pluripotent stem cell, HIV-associated neurocognitive disorder, HIV encephalitis, latent reservoir

## Abstract

The central nervous system (CNS) is a major human immunodeficiency virus type-1 (HIV-1) reservoir. Microglia are the primary target cell of HIV-1 infection in the CNS. Current models have not allowed the precise molecular pathways of acute and chronic CNS microglial infection to be tested with *in vivo* genetic methods. Here, we describe a novel-humanized mouse model utilizing human induced pluripotent stem cell (iPSC)-derived microglia to xenograft into murine hosts. These mice are additionally engrafted with human peripheral blood mononuclear cells that serve as a medium to establish a peripheral infection that then spreads to the CNS microglia xenograft, modeling a trans-blood-brain barrier route of acute CNS HIV-1 infection with human target cells. The approach is compatible with iPSC genetic engineering, including inserting targeted transgenic reporter cassettes to track the xenografted human cells, enabling the testing of novel treatment and viral tracking strategies in a comparatively simple and cost-effective *in vivo* model for neuroHIV.

**IMPORTANCE:**

Our mouse model is a powerful tool for investigating the genetic mechanisms governing central nervous system (CNS) human immunodeficiency virus type-1 (HIV-1) infection and latency in the CNS at a single-cell level. A major advantage of our model is that it uses induced pluripotent stem cell-derived microglia, which enables human genetics, including gene function and therapeutic gene manipulation, to be explored *in vivo*, which is more challenging to study with current hematopoietic stem cell-based models for neuroHIV. Our transgenic tracing of xenografted human cells will provide a quantitative medium to develop new molecular and epigenetic strategies for reducing the HIV-1 latent reservoir and to test the impact of therapeutic inflammation-targeting drug interventions on CNS HIV-1 latency.

## INTRODUCTION

The pandemic of human immunodeficiency virus type 1 (HIV-1) causes a chronic infection without a known universal cure (1). While there have been several reported cases of HIV-1 remission and cure after bone marrow and cord blood transplantations (2), these procedures are extremely high risk and have been developed for rare cases of patients with life-threatening, co-morbid leukemia (3). A significant challenge to find a sterilizing cure has been the persistence of latently infected cellular reservoirs that resume replication when antiretroviral therapy (ART) is withdrawn (4–9). Furthermore, while ART can suppress viremia to undetectable levels, people with HIV-1 (PWH) experience more premature aging and inflammation-associated pathophysiology than uninfected people (10–14). The central nervous system (CNS) carries a heavy disease burden with HIV-1-associated neurocognitive disorder affecting 20%–50% of PWH (15–18).

HIV-1 enters the brain during the first initial weeks of acute infection (19–22) via transmigration of HIV-1-infected cells across the blood-brain barrier (BBB) (23–25). In the periphery, HIV-1 targets the CD4^+^ T-cells and monocytes that then disseminate infection to other tissues and organs (23–28). In the CNS, these peripherally infected cells primarily target microglia comprising about 5%–10% of adult brain cells (29). Our recent studies on the human postmortem brain corroborate that HIV-1 infection occurs predominantly in microglia, and provirus integration is linked to inflammation-associated reprogramming of microglial transcriptomes and 3D genomes (15). Understanding the molecular mechanisms underlying the establishment and maintenance of actively and latently HIV-1-infected microglia in the CNS will help investigate ways to target and reduce CNS reservoirs and develop therapeutic interventions for microglia-associated neuroinflammation.

Genetic animal models that capture the salient features of CNS HIV-1 infection in humans are greatly needed to interrogate the precise mechanisms governing the HIV-1 life cycle in the host. These can enable the study of initial infection of the peripheral immune system, viral replication, dissemination throughout all organ systems, including the CNS microglia and other myeloid compartments, and provirus integration into the host cell genome to maintain chronic HIV-1 infection. To this end, elegant non-human primate models using various strains of simian immunodeficiency virus have long been established in the field (30, 31). However, these models are still inherently limited, given that HIV-1 is a human-specific retrovirus (32).

Although HIV-1 does not replicate in mouse immune cells, the repertoire for modeling neuroHIV has been expanded by employing chimeric virus models to infect mice. The EcoHIV model utilizes a chimeric murine-tropic virus that has been genetically modified to carry a murine leukemia virus envelope coding region and, hence, can infect and simulate HIV-1 disease-like phenotype in mice (33). To study native HIV-1 in small animal models, investigators have employed human immune cell xenografted immunodeficient mouse models. While initially, studies focused on blood and peripheral systems (34, 35), a recent advance has enabled CNS engraftment of human cells by using human fetal hematopoietic tissue or cord blood-derived CD34^+^ hematopoietic stem cells (HSC) into immunocompromised NOG mice expressing a human interleukin (IL)-34 transgene. These mice show colonization of multiple lymphoid and myeloid compartments, including differentiation of microglia-like brain cells susceptible to HIV-1 infection (36, 37). Since then, another mouse model, where human IL-34 transgene expressing NOG mice are xenografted with human fetal liver tissue, fetal thymic tissue, and liver-derived CD34^+^ hematopoietic stem and progenitor cells (HSPC), was reported to undergo human microglia reconstitution in CNS that are susceptible to HIV-1 infection (38). Other models rely on the direct engraftment of HIV-1-infected human myeloid cells into the brain of immunocompromised mice (39).

These humanized mouse models have been extremely valuable for expanding knowledge on molecular and cellular signatures of HIV-1-infected brains. However, currently existing humanized mouse models also pose some limitations. These include the requirement of human fetal tissue or umbilical cord blood that is limited in supply and not readily amplified to conduct extensive tests with the same genotype (36, 38, 40) and exhibit a lower rate of xenograft reconstitution following irradiation or chemotherapy (36–38) as compared to induced pluripotent stem cell (iPSC)-derived cells. Some of these models also feature a non-physiological intracranial route of HIV-1 inoculation into the brain (36, 39, 41). Here, we present a novel humanized mouse model for HIV-1 infection that implements genetically engineered human iPSCs as a near-unlimited resource to reconstitute neonatal mouse brains with human microglia. The unrivaled versatility of iPSCs enables genetic material to be clonally engineered and provides an invaluable toolbox for generating reporter cell lines. Furthermore, in our model, CNS-engrafted mice are peripherally engrafted with human peripheral blood mononuclear cells (huPBMC) in early adulthood to initiate infections from peripheral vasculature. Infected huPBMCs then travel to the CNS to infect the xenografted microglia (xenoMG). Mice dually engrafted with xenoMGs and huPBMCs are infected with M-tropic HIV-1. This models a physiological route of acute CNS HIV-1 infection through virus or infected cells crossing the BBB.

## RESULTS

### Differentiation of microglia from genetically modified human iPSC *in vitro*

Studying CNS HIV-1 infection has been challenging due to the limited accessibility of human samples and difficulty in tracing persistently infected cellular reservoirs, even with suppressive ART. This study presents a novel human iPSC-based cell lineage tracing model that irreversibly marks HIV-1-infected cells for subsequent investigation. The iPSC is an ideal model due to its potential to differentiate into multiple cell types and to generate clonal lines (42, 43). Specifically, we chose the WTC11 iPSC as our parental line for the following reasons: (i) WTC11 can be robustly differentiated into different cell fates, including microglia-like cells (44, 45); (ii) WTC11 possesses a well-known stable 46XY karyotype that is highly conducive to gene editing (to date, over 60 different gene-edited WTC11 lines have been generated and are available through the Allen Cell Collection [https://www.allencell.org/cell-catalog.html]; and (iii) the complete genome sequence of the WTC11 line is publicly available, greatly facilitating the design of gene editing experiments.

We genetically modified the WTC11 line by introducing a Cre-recombinase-dependent, CAG promoter-driven dsRed-to-eGFP switch cassette into the AAVS1 locus within the PPP1R12C gene (MSE2104 iPSC line, Fig. S1A). We considered other potential safe harbor loci, including human Rosa 26 (hROSA26) (46), CCR5 (47), and CLYBL (48). We ruled out the CCR5 locus because of its association with HIV-1 biology. Additionally, we excluded the CLYBL locus because we observed significant silencing effects during differentiation into HSCs (unpublished data). Our focus shifted to AAVS1 because of its widespread utility in the iPSC field. The AAVS1 locus is also ideal because it permits robust, stable, and reproducible expression of transgenes across multiple lineages maintained long term in culture (49). Furthermore, it mitigates pleiotropic position effects, ensuring that the inserted transgene is not silenced during meso- and ectoderm differentiation. In our transgenic MSE2104 iPSC line, the genetic switch is encoded by inserting a dsRed coding sequence followed by a stop codon into a loxP Cre-recombinase target site upstream of an eGFP coding sequence. This configuration enables Cre-dependent switching from dsRed to eGFP (Fig. S1B and C).

**Fig 1:**
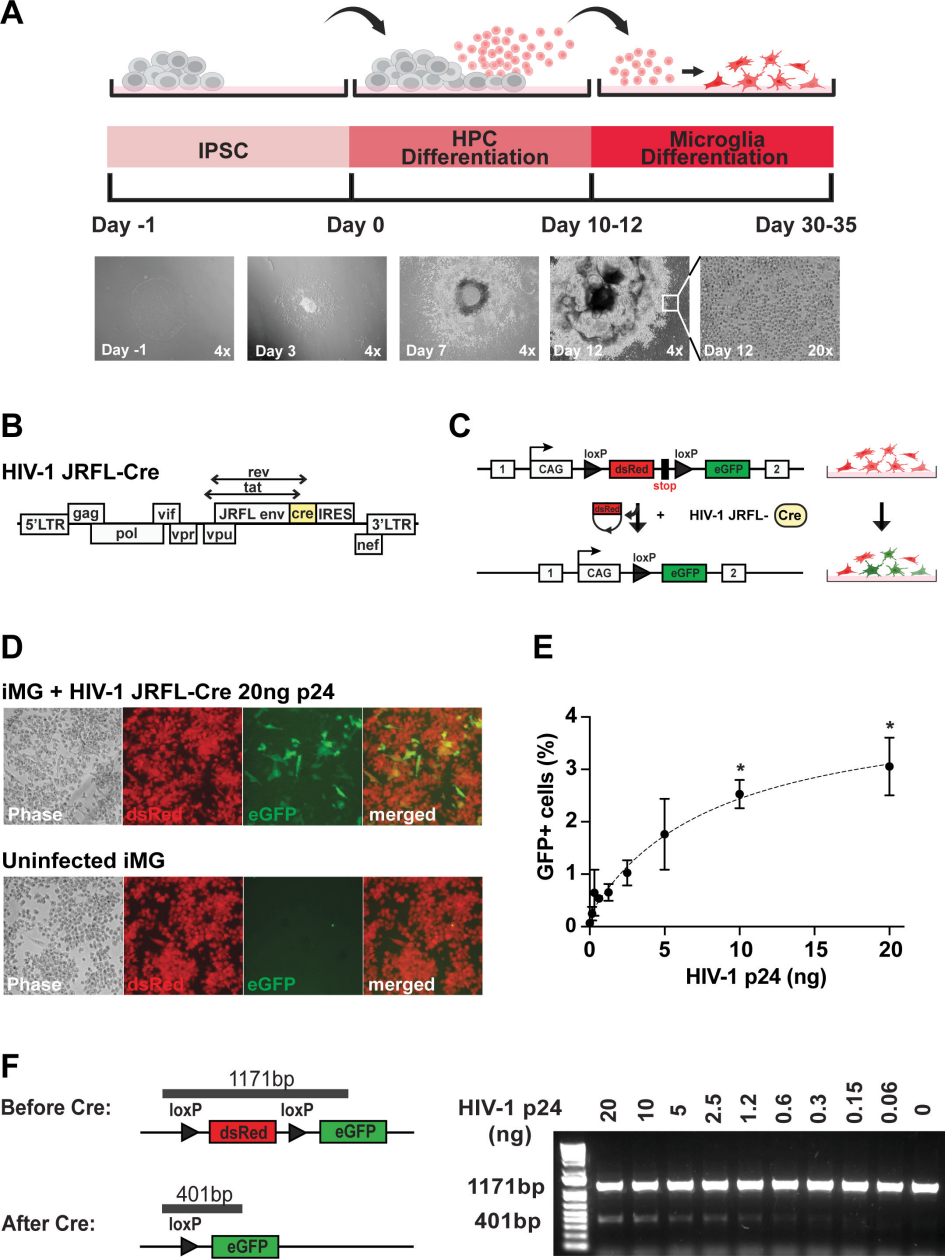
Overview of *in vitro* iPSC experiments. (**A**) (Top) Timeline of cellular differentiation of the MSE2104 iPSC-HPC-iMG line harboring the conditional fluorescent reporter transgene at the AAVS1 locus (refer to Fig S1A). (Bottom) Corresponding stage-specific morphological appearances of cell cultures at low power magnification (4×–20×, as indicated). (**B**) Schematic of the M-tropic JFRL-Cre HIV-1 genome, including Cre coding cassette followed by IRES to drive Nef expression. This viral clone facilitates the Cre-dependent conditional recombination of the dsRed-to-eGFP reporter. (**C**) Schematic of dsRed-to-eGFP color switch observed in MSE2104 iPSC-derived microglia (iMG) infected with HIV-1 JRFL-Cre. (**D**) (Top) Conditional eGFP transgene expression in a subset of HIV-1 JRFL-Cre-infected iMG, and (bottom) absence of eGFP expression in uninfected iMG. Cells were infected with 0–20 ng HIV-1 p24 for 48 h. Quantification of HIV-1 JRFL-Cre-infected iMG demonstrated (**E**) a maximum of 3% HIV-1 infection efficiency in cells infected with the highest amount (20 ng) HIV-1 p24. Error bars are SEM of *n* = 3. The asterisk (*) represents statistical significance (*P* < 0.05) on a two-sample *t*-test comparing HIV-1 infected vs uninfected iMG sample. (**F**) Cre-mediated dsRed deletion generates a shorter 401 bp recombinant PCR product. HIV-1 p24 dose-dependent (0–20 ng) infection and subsequent Cre-loxP recombination is observed.

The iPSCs were differentiated into hematopoietic progenitor cells (HPC) using a commercially available STEMdiff Hematopoietic kit as per published protocols (Fig. 1A) (44, 50–53). The development of HPC from iPSCs requires a combination of growth factors, cytokines, and small molecules, which include stem cell factor, Flt3 ligand, thrombopoietin, IL-3, IL-6, and granulocyte macrophage colony-stimulating factor (54, 55). Differentiating iPSC into HPC took 10 days, after which cells were harvested between days 10 and 12 for downstream experiments. We validated our HPC differentiation by measuring established HPC-specific CD34, CD43, and CD45 cell surface marker expression on flow cytometry. As expected, more than 90% of HPC expressed CD34 and CD43, and approximately 50%–60% of HPC expressed CD45 (Fig S2A) (44, 50, 51, 56).

To generate homeostatic microglia (iPSC-derived MG, or iMG) *in vitro*, HPCs were treated with cytokines IL-34, transforming growth factor beta 1, and macrophage colony-stimulating factor (M-CSF) (Fig. 1A) (44, 50–53, 56). Differentiation of HPC to iMG was followed for 20–25 days, and phenotypes were validated by measuring the surface expression of common myeloid marker CD45 and microglial markers, Iba1 and P2YR12 (Fig S2B). Macrophage-specific CD206 and monocyte-specific CD14 cell surface markers were examined to distinguish our iMGs from macrophages and monocytes. More than 90% of our iMGs expressed CD45 and CD11b, and approximately 80% expressed P2YR12 (Fig S2B). Furthermore, most of our WTC11 iPSC-derived iMGs stained positive for CD45, Iba1, and P2YR12 on immunofluorescence (Fig S2C).

### *In vitro* HIV-1 susceptibility of microglia derived from Cre-activated fluorescent reporter iPSC

Next, we tested whether *in vitro* differentiated iPSC-derived cells could sustain HIV-1 infection. We constructed an HIV-1 clone that carries the M-tropic JRFL envelope to target all myeloid lineage cells, including microglia (Fig. 1B) (57–59). We selected the HIV-1 JRFL clone for its known neurotropism, specifically for myeloid lineage cells, including microglia (60–62). The HIV-1 JRFL was first isolated from the frontal lobe of a patient with HIV-1-associated dementia (60, 62). Moreover, studies have demonstrated myeloid lineage cell-specific fusogenic potential of the HIV-1 JRFL (59, 63). This virus also expressed Cre instead of the viral Nef, an early viral gene expression indicator. Cre-recombinase-dependent cellular changes occurred only after HIV-1 viral integration (64). Nef expression was restored by inserting an internal ribosome entry site (IRES) upstream of the Nef open reading frame, as previously described (64). Inserting the IRES to restore Nef activity is an efficient strategy for expressing heterologous genes in the context of replication-competent HIV-1 (65). Analogous fluorescent protein-expressing, IRES-carrying viral clones are infectious, capable of mediating high viremia and CD4^+^ T-cell depletion *in vivo* in humanized mice (66).

The iMGs were infected with HIV-1 JRFL-Cre, as previously described (59, 64, 66, 67). After 48 h, HIV-1 JRFL-Cre-infected cells started to express eGFP (Fig. 1C and D). On average, we saw approximately 3% of HIV-1-infected cells (Fig. 1E). Many iMG cells continued to express dsRed. Some infected cells even appeared to co-express dsRed and eGFP (Fig. 1D). This dsRed and eGFP co-expression by infected iMGs is likely because eGFP is expressed early upon provirus integration. Dsred has a long maturation time and half-life (68, 69). The Cre recombinase-mediated deletion of dsRed in infected iMGs was confirmed by DNA PCR (Fig. 1F). Hence, our fluorescent reporter iPSC line can be successfully used to generate human microglia that could serve as a tool for studying HIV-1 infection at a single-cell level *in vitro* and, as described below, *in vivo*.

### A novel-humanized mouse dually xenografted with human iPSC-derived microglia and huPBMC

**Fig 2:**
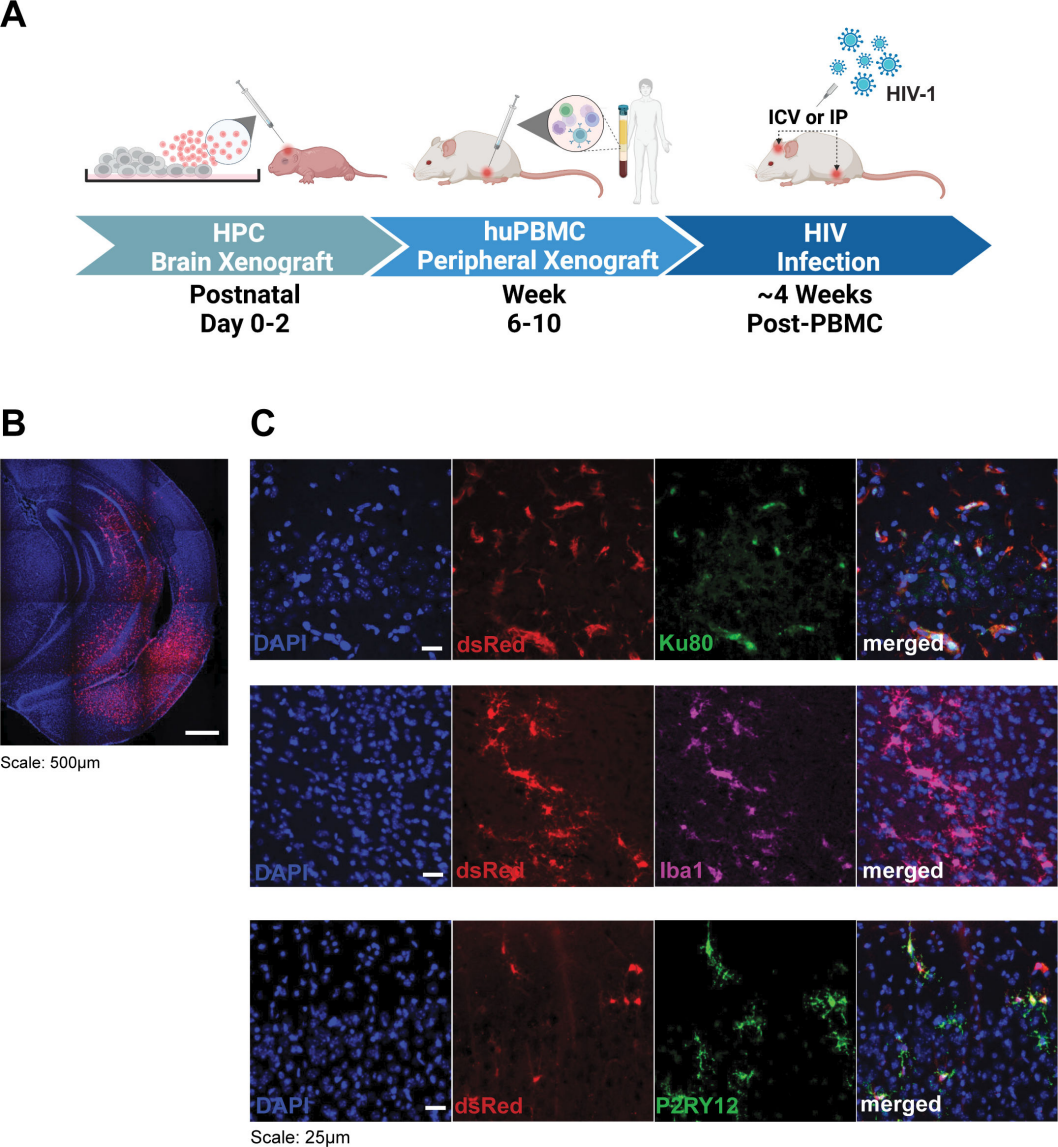
Humanized mouse model with central xenoMG and peripheral huPBMC engraftment. (**A**) Timeline of the dual engraftment process and HIV-1 infection of humanized mouse. MSE2104 iPSC-derived HPCs, or precursors of microglia xenograft (xenoMG), were intracranially injected into neonatal mouse brains upon birth between days 0 and 2. Mice were subsequently injected with huPMBC intraperitoneally between weeks 6 and 10. The mice were then infected with HIV-1 about 4-week post-huPBMC injection via one of two routes: ICV or IP. (**B**) The whole hemisphere view of DAPI (blue) counterstained coronal brain section of a 2-month-old mouse neonatally injected with HPCs (at 4× magnification). A diffuse spread of dsRed+ cells is observed at particularly high densities in the medial temporal lobe, including in the hippocampus. (**C**) Immunohistochemistry on coronal brain sections of the above mouse at 40× magnification. Layers I and/or II of the adult mouse cerebral cortex demonstrate colocalization of dsRed+ cell signal with human-nuclei-specific Ku80 and microglia-specific markers, Iba1 and P2RY12. All sections were counterstained with DAPI (blue).

Animal models for studying CNS HIV-1 infection have been limited in elucidating the precise mechanisms governing CNS dissemination and establishing chronic viral reservoirs *in vivo*. Here, we present a novel mouse model xenografted with iPSC-derived microglia that enables tracing of HIV-1-infected cells at a single-cell resolution and, hence, could shed more light on the HIV-1 life cycle related to CNS HIV-1 infection. We use immunocompromised mice harboring the human MCSF (CSF1) knock-in allele in a Rag2 and Il2rγ knockout background to facilitate successful central engraftment of human microglia. These mice express at least one allele of the human *CSF1* gene that critically supports the development of human iPSC-derived microglia in the mouse brain (44, 50–53, 70). Mice lacking *Rag2* and *Il2r*γ genes have no native T-cells, B-cells, or NK cells, making them ideal hosts for xenografted human cells. Genetically modified fluorescent reporter iPSC described above were differentiated into HPC and injected intracranially into newborn pups on postnatal days 0–2, as previously described (44) (Fig. 2A).

To confirm that our genetically engineered iPSC reporter cells colonize the mouse brain as xenografted microglia (xenoMG), we collected the brains of mice (*n* = 6) that were centrally injected with iPSC-derived HPCs at 8 weeks of age. We observed dsRed-expressing xenografted cells in the cerebral cortex of all six injected mice forebrain. Figure 2B shows a representative coronal section of a mouse brain colonized with dsRed+ cells. To verify that the engrafted dsRed+ cells are human and *in vivo* differentiated microglia, we immunohistochemically labeled xenografted mouse brain sections with human-specific nuclear antigen, Ku80 (44), and microglia-specific cell marker, ionized calcium-binding adapter molecule 1 (Iba1) and purinergic receptor P2RY12, antibodies. Indeed, the dsRed+ cells colocalized with Ku80, Iba1, and P2RY12 cell markers (Fig. 2C), confirming that the centrally injected iPSC-derived HPC differentiated into xenoMG.

To develop a model system whereby infection of peripheral immune cells spreads to the CNS, xenoMG mice engrafted with iMG at birth were additionally engrafted with huPBMCs at 6–10 weeks of age. In this model, a route that transits the blood-brain-barrier may be achieved by intraperitoneal (IP) inoculation rather avoiding the need for direct (non-physiological) injection of virus intracerebroventricularly (ICV) (Fig. 2A). This mimics aspects of a physiological route of acute CNS HIV-1 infection in people, where peripheral CD4^+^ T-cells or monocytes are the initial HIV-1 targets that are then trafficked to the brain to infect microglial cells, which are the primary resident HIV-1 target in the CNS. Dual-engrafted mice were IP injected with *in vitro* activated huPBMCs. Human leukocytes that populate the mice are predominantly memory T-cells as monitored by human leukocyte-specific cell surface markers: leukocyte common antigen CD45 and T-cell markers CD3, CD4, and CD8 (Fig S3) and are highly susceptible to HIV infection (71).

### *In vivo* HIV-1 infection of xenoMG in humanized mouse brain

Until recently, previously described humanized mouse models for CNS HIV-1 infection were largely limited to a non-physiological route of infection by directly injecting infected cells, or the HIV-1 virus, into the brain (36, 38, 72). This limitation is due to the inherent tropism of HIV-1 for human immune cells and with the understanding that mouse peripheral immune cells are resistant to HIV-1 infection and, thus, cannot transmit the infection to the CNS unless dually engrafted with human peripheral immune cells. We, therefore, conducted a direct side-by-side comparison of two different modes of infection for neuroHIV: ICV (73) vs IP.

Additionally, we examined whether peripheral engraftment of human immune cells is necessary for HIV-1 infection of central xenoMG when these mice are infected peripherally. To test the requirement of peripheral human immune cell engraftment, mice pups (*n* = 18) that were centrally injected with xenoMG precursors were randomized into two groups at 3 months. Group 1 included mice that were dually engrafted with xenoMG in the brain and with huPBMC in the peripheral blood (*n* = 6) (Fig. 2; Fig. S4), and Group 2 included mice that were singly engrafted with xenoMG in the brain (*n* = 12) (Fig S4). In Group 1, three mice were ICV-infected, and three were IP-infected with HIV-1 JRFL-Cre. In Group 2, two mice were ICV-infected, and two were IP-infected with HIV-1 JRFL-Cre. Eight remaining mice in Group 2 were used as uninfected control mice.

The six dually engrafted mice in Group 1 reached a median of 20% (range: ~10%–30%) huPBMC engraftment at about 4 weeks post-injection when the mice reached about 3 months of age. Group 1 mice were randomized to two modes of HIV-1 infection, ICV and IP. Peripheral HIV-1 infection was monitored weekly by HIV-1 viral qRT-PCR from mouse cheek blood. The dually engrafted (huPBMC and xenoMG) Group 1 mice had measurable viremia, with HIV-1 RNA levels 2–3 orders of magnitude above the detectability threshold as compared to the singly (xenoMG) engrafted Group 2 mice, regardless of the route of infection (Fig S4B). The HIV-1 viral copy number in the Group 1 huPMBC engrafted mice ranged at 10^1^–10^3^ copies of viral RNA transcript per mL plasma (Fig S4B). This would be comparable to early stages of HIV-1 infection in human subjects whose average viral load, if left untreated, could reach 30–50 × 10^3^ copies/mL before initiating effective antiretroviral therapies (ART) (74–76).

**Fig 3:**
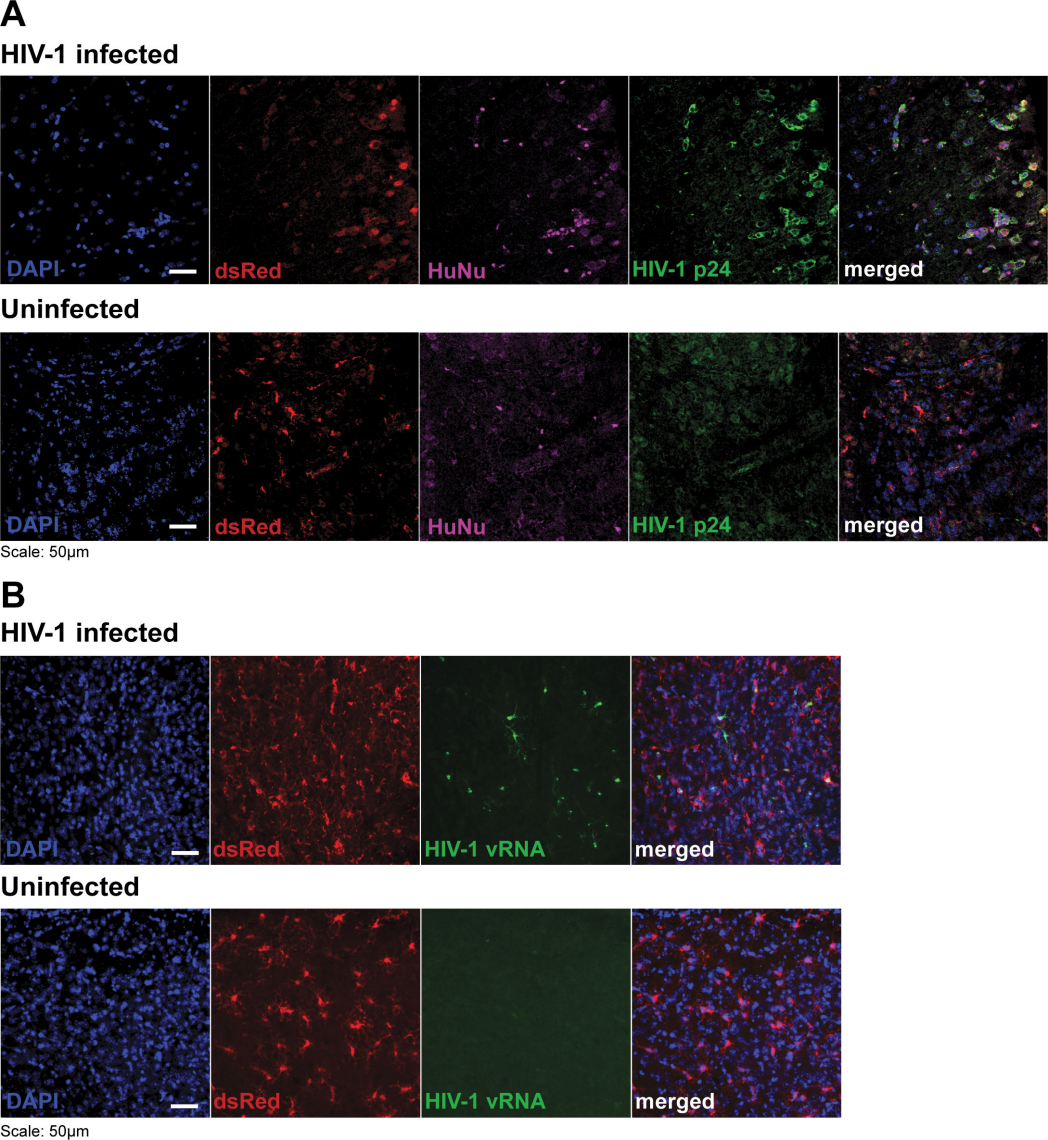
MSE2104 iPSC-derived xenoMG is infected with HIV-1 via the peripheral route. (**A**) Immunohistochemistry detected the presence of HIV-1 p24 antigen-antibody in representative adult mouse brain cortical sections (*n* = 4 HIV-1 infected, *n* = 4 uninfected mice) that were xenografted with dsRed-expressing MSE2104 iPSC-derived microglia (xenoMG). Mice were peripherally engrafted with huPBMC, after which they were intraperitoneally infected with HIV-1. All brain sections demonstrated dsRed + xenoMG that were counterstained with DAPI (blue). Human nuclear antigen (HuNu, magenta) antibody-marked human cells co-stained with HIV-1 p24 (green) in (top) HIV-1 infected and (bottom) uninfected. Note microglial nodule-like structure in bottom right quadrant of section from HIV-1 infected brain (see text). (**B**) HIV-1 gag-pol RNAScope FISH demonstrated HIV-1 vRNA expression (green) in only (top) HIV-1-infected mouse brain xenoMG and not in (bottom) uninfected mouse brain xenoMG.

We next IP-infected a third cohort of dually engrafted mice (Group 3, *n* = 30) with HIV-1 JRFL-Cre. Here, we found that peripheral viremia peaked around week 3 or 4 (Fig S5; Table S1) when the brains were collected. A subset of mouse brains (*n* = 8) was for histological analysis to verify central HIV-1 infection. The brain sections of HIV-1-infected dually engrafted mice revealed human nuclear antigen positive (HuNu+) cell nuclei surrounded by a halo of HIV-1 p24 antigen in the cytoplasm, confirming successful infection (Fig. 3A). Remarkably, a subset of HIV-1 p24+ cells presented as large multinucleated HuNu+ cells (Fig. 3A arrowheads), reminiscent of the well-described multinucleated microglial nodules of HIV-1-infected cells in the encephalitic human brain, particularly in cases with severe neuroinflammation and encephalitis (77, 78). In addition, cellular morphologies of xenoMGs from HIV-1-infected mouse brains included many cells with amoeboid shape and enlarged somata with fewer and shorter and wider processes reminiscent of similar findings in clinical specimens (77, 79, 80) (Fig. 3A). For additional confirmation of successful xenoMG infection with HIV-1 JRFL-Cre, we performed RNAScope fluorescence *in situ* hybridization (FISH) using HIV-1-specific oligonucleotide probe. There was robust HIV-1 viral RNA (vRNA) expression in the xenoMGs of HIV-1-infected mouse brains. In contrast, the brains of uninfected mice did not demonstrate any signal above the background (Fig. 3B).

### Infection rate and immunological response of xenoMG linked to peripheral HIV-1 viremia

**Fig 4:**
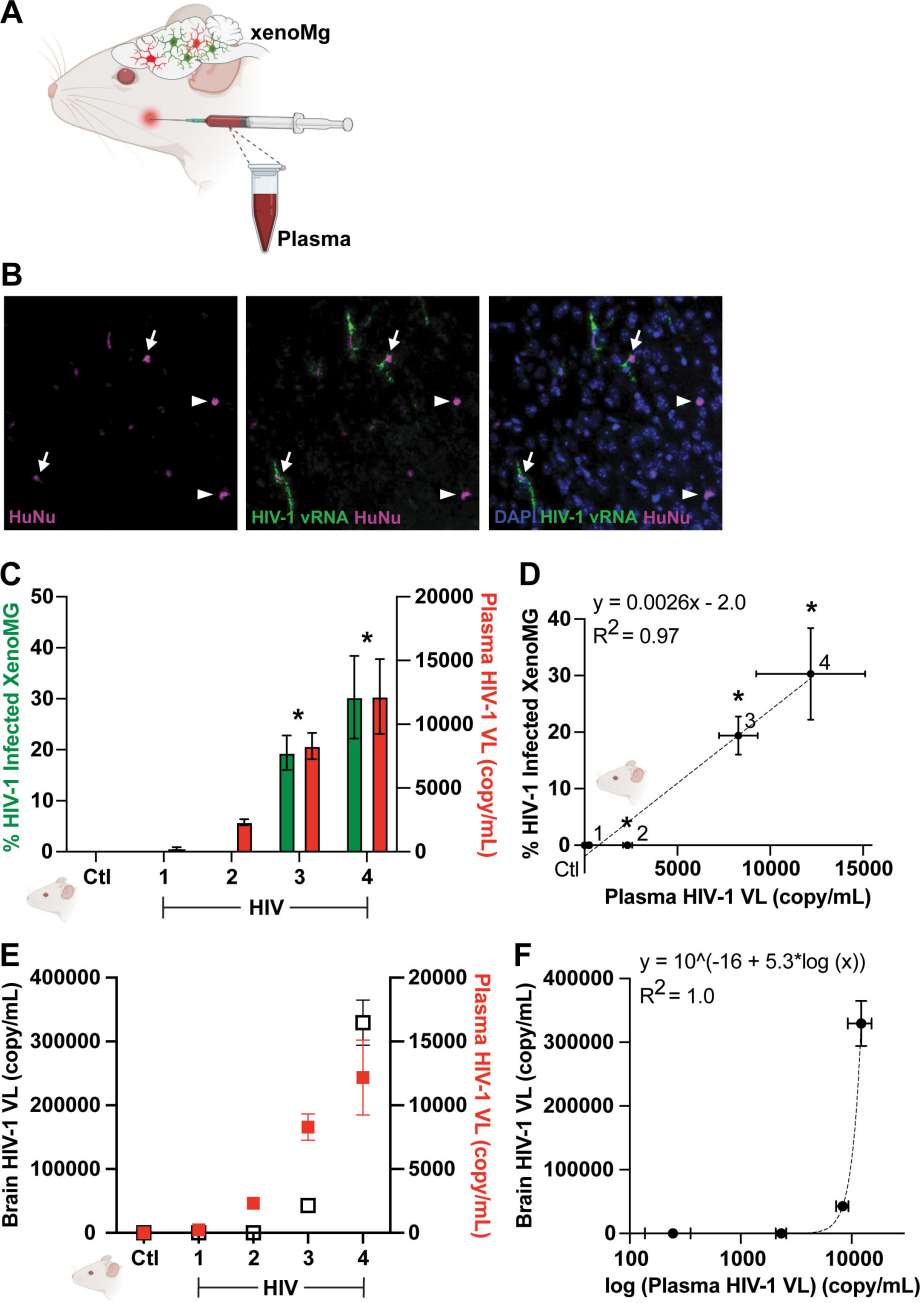
Percentage of HIV-1-infected xenoMG is proportional to HIV-1 viremia in the plasma and brain of dually xenografted humanized mice. (**A**) Schematic of tissue samples collected from representative HIV-1-infected (*n* = 4) and uninfected (*n* = 1) mice dually engrafted with xenoMG centrally and huPBMC peripherally. Cortical brain sections of the left hemisphere of the mouse brain were used for staining with HuNu antibody and HIV-1 gag-pol RNAScope FISH. The right hemisphere was used for total RNA extraction to measure central HIV-1 viremia. Cheek blood was collected to isolate plasma to measure peripheral HIV-1 viremia. (**B**) A representative section from an HIV-1-infected mouse demonstrates HuNu+ cells (magenta) that are HIV-1 infected (arrow), as demonstrated by co-expression of HIV-1 vRNA (green), as well as HuNu+ cells that are uninfected (arrowhead) and, hence, do not express HIV-1 vRNA. (**C**) The percentage of HIV-1 vRNA+ xenoMG (left *y*-axis, green) was proportional to the plasma HIV-1 viral load (copy/mL) (right *y*-axis, red) in HIV-1-infected mice (#1–4 on the *x*-axis) and uninfected control mouse (Ctl on the *x*-axis). The asterisk (*) above the bar graphs represents statistical significance (*P* < 0.05) on a two-sample *t*-test comparing individual HIV-1-infected mice to the uninfected control mouse. (**D**) Linear correlation between the percentage of HIV-1-infected xenoMg and complementary plasma HIV-1 viral load. The dotted line represents a fitted linear graph of data points with a significant correlation constant (*R*^2^) of 0.97. (**E**) Brain HIV-1 viral load (left *y*-axis, white) is plotted with corresponding plasma HIV-1 viral load (right *y*-axis, red) in HIV-1-infected mice (#1–4 on the *x*-axis) and uninfected control mouse (Ctl on the *x*-axis). (**F**) Exponential correlation (*R*^2^ = 1) between HIV-1 viral load in the plasma and brain. All error bars in this figure represent the SEM of at least three replicates.

We next measured the extent of HIV-1 infection in xenoMG of mouse brain sections. We dually stained the brain sections with HIV-1-specific oligonucleotide probe for fluorescent *in situ* hybridization (FISH) and with HuNu antibody for immunohistochemistry. We focused on five representative mice for quantification: four dually engrafted, IP-infected mice (*n* = 4) and one dually engrafted, uninfected control mouse (*n* = 1). We calculated the ratio of HIV-1-infected xenoMGs to uninfected cells in these mouse brain sections and measured the peripheral HIV-1 viral load in the mouse plasma (Fig. 4A). The HIV-1-infected xenoMGs were defined as those that were double-positive for HIV-1 vRNA and HuNu (Fig. 4B). We observed xenoMG infection rate from as low as <10% up to 40% in the dually engrafted mouse brain sections (Fig. 4C). There was a significant correlation (*R*^2^ = 0.97) between the percentage of HIV-1-infected xenoMG in the brain and terminal plasma HIV-1 viremia level (Fig. 4C and D). The mouse with the highest plasma viral load (1.2 × 10^4^ HIV-1 copies /mL plasma) reached an average of ~30% infection rate in the xenoMG. We also observed that the terminal plasma HIV-1 viremia level significantly correlates (*R*^2^ ≈ 1) with brain HIV-1 viral load (Fig. 4E and F).

**Fig 5:**
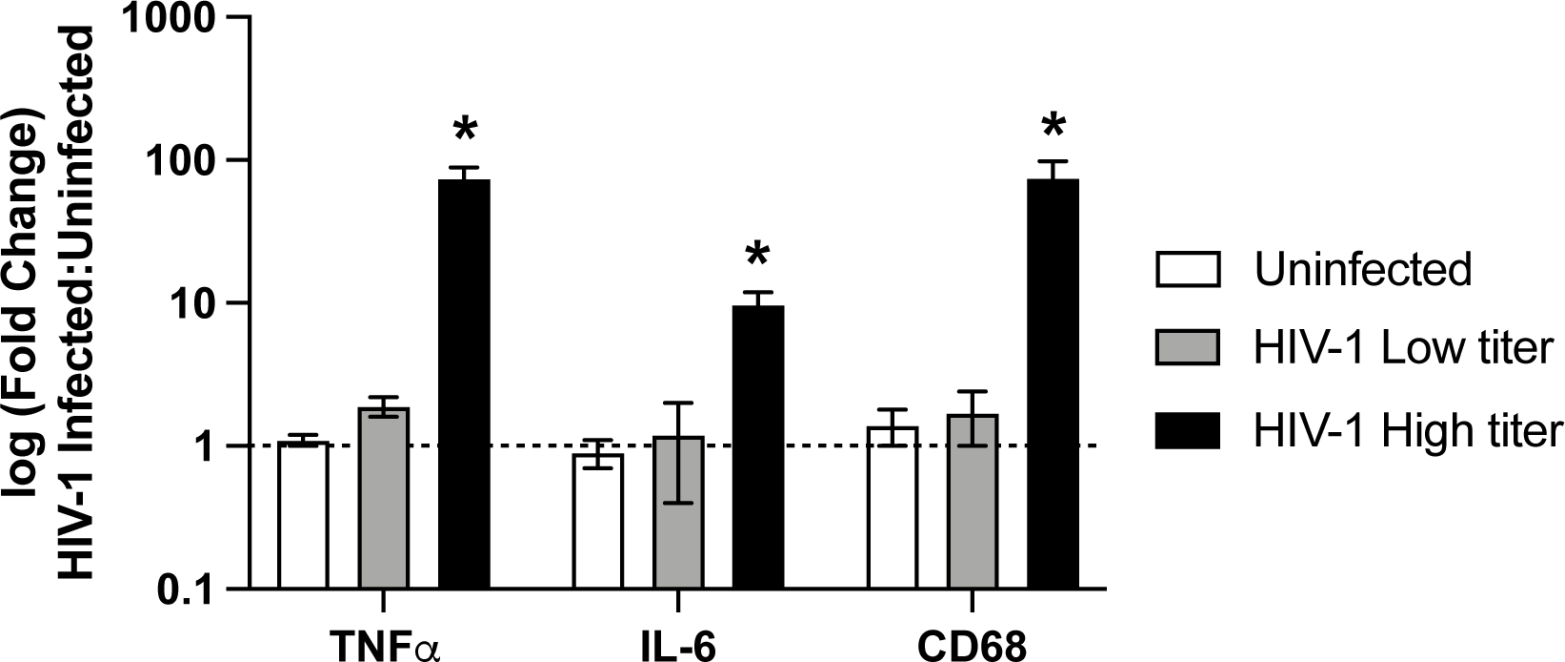
HIV-1-infected dually xenografted humanized mice exhibit a heightened proinflammatory response in the brain. Quantitative qRT-PCR of three proinflammatory markers, TNFα, IL-6, and CD68 (*x*-axis), in the brains of HIV-1-infected humanized mice using human-specific Taqman gene expression probes. Bar graphs represent fold change in HIV-1-infected mice (*n* = 4) compared to uninfected control mice (*n* = 4). Two mice had low-liter (<10 copies) and two mice had high-titer (>10^5^ copies) HIV-1 viremia. The asterisk (*) above the bar graphs represents statistical significance (*P* < 0.05) on a two-sample *t*-test comparing HIV-1-infected mice to uninfected mice.

It is well-established in the literature that chronic HIV-1 infection is linked to proinflammatory responses underlying clinical comorbidities, including HIV-1-associated neurocognitive disorder (15–18). Hence, we wondered whether HIV-1-infected xenoMG exhibits higher proinflammatory cytokine expression than an uninfected mouse brain cell. To test this notion, we measured the expression of two proinflammatory cytokines, tumor necrosis factor alpha (TNFα) and IL-6, and the microglial activation marker CD68 in mouse brain tissue extracts by performing qRT-PCR using human-specific primers (81–83) (Fig. 5). As expected, the HIV-1-infected mouse brains (*n* = 4) expressed higher levels of TNFα, IL-6, and CD68 transcript compared to uninfected mouse brains (*n* = 4) (Fig. 5). Moreover, the proinflammatory marker expression significantly correlated with the HIV-1 viral load of terminally collected brain tissue (*P* < 0.05 on 2-sample *t*-test using HIV-1 infected vs uninfected brain samples) (Fig. 5). Mouse brain with low HIV-1 vRNA copies (defined as <10 copies per 100 ng total RNA) had lower expression of proinflammatory markers compared to mice with higher HIV-1 vRNA copies (defined as >10^5^ copies per 100 ng total RNA) (Fig. 5). Hence, the HIV-1-infected mouse exhibited higher proinflammatory response in the brain compared to what was observed in the uninfected mouse brain, and the level of response was significantly associated with the degree of HIV-1 viral transcription.

## DISCUSSION

This study presents a novel-humanized mouse model in which mice are dually engrafted centrally with human iPSC-derived and genetically engineered microglia and peripherally with huPBMC. While these mice can be infected with HIV-1 in the brain via ICV or IP injection, as demonstrated above, the peripheral route offers a significant advantage in modeling the typical route of CNS infection in human cases. A major strength of our model lies in the remarkable versatility of iPSC, including their potential for use in genetic engineering. This includes integrating a transgenic reporter system that enables us to track the transplanted cells over an extended period, up to 4 months after transplantation into the neonatal mouse brain.

The differentiation of iPSCs into microglia, followed by their xenografting into the mouse brain, provides an abundant and renewable source of microglia. This approach also allows for genetic modification before transplantation and subsequent *in vivo* experimentation, thus facilitating a more intricate understanding of CNS HIV-1 pathogenesis. Genetically modified stem cells have proven immensely valuable in HIV-1 research. It enables the evaluation of cellular susceptibility and resistance to HIV-1 infection by altering viral docking sites on the cell surface (84), as well as exploration of cell type-specific transcriptional responses to infection and antiretroviral treatment in a tightly controlled, isogenic environment (85, 86). Despite the critical importance of these molecular and mechanistic studies, they have primarily been conducted *in vitro* or *ex vivo*, which can limit their clinical relevance.

Our novel-humanized mouse model presents a promising alternative for preclinical and translational research *in vivo*. It serves as a valuable platform for assessing genetically engineered human iPSC-derived cells within the brain, thus paving the way for developing more effective therapies targeting microglia-related HIV-1-associated CNS comorbidities, such as HIV-1-associated neurocognitive disorder (15). Moreover, our model has the potential to generate other unconventional cell-based humanized mouse models for studying HIV-1, including those involving astrocytes. The role of astrocytes in HIV-1 infection and their contribution to viral reservoirs with clinical implications lack a consensus (83, 87). Even in the context of end-stage HIV-1 encephalitis, astrocytes harbor only a tiny proportion of HIV vRNA+ cells, accounting for only 0.3%–1% of the total astrocyte population (15, 83); consequently, while our model system likely has the potential to generate iPSC-derived astrocyte-humanized mouse model, similar to the approach presented here for microglia, the significance of this model remains to be tested.

To develop novel therapies for treating and potentially eradicating HIV-1, we propose using genetically edited iPSCs targeting promising genes. For example, the CCR5 Δ32 mutation has shown significant promise as a curative target against HIV-1, as demonstrated by multiple clinical studies indicating its high resistance to the virus (88–92). Similarly, SAMHD1, a host restriction factor that impedes HIV-1 reverse transcription in myeloid cells, represents a potential therapeutic target for combating HIV-1 (93–97). Consequently, our genetically engineered iPSC-based mouse model significantly broadens the repertoire of humanized mouse models for studying CNS HIV-1 infection. This expansion in model diversity amplifies our capacity to explore innovative approaches to combat this debilitating disease.

Several other humanized mouse models have been developed to study CNS HIV-1 infection (36–38, 72, 98–100). The hIL34-NOG model involves intrahepatic transplantation of umbilical cord blood-derived CD34^+^ HSPCs that differentiate into human microglia in mouse brains over 6–8 months before infection with HIV-1 (36, 37, 72). The hu-BLT-hIL34-NOG is a modification of the hIL34-NOG model that involves transplantation of human fetal liver, thymic tissue, and fetal liver-derived CD34^+^ HSPCs. This model reconstitutes the human immune system in the mouse by 16 weeks of age, and the mouse is susceptible to HIV-1 infection (32, 38, 41). However, these models are limited by their restricted options for genetic manipulation. Our iPSC-based humanized mouse model allows clonal expansion of genetically modified iPSCs with uniform penetrance of the altered gene. This holds tremendous potential for generating knock-out, knock-in, or conditional transgenes to definitively examine the role of genes within an *in vivo* context.

In addition, both models require 4–5 months for the complete reconstitution of the human hematolymphoid system before effective HIV-1 infection can occur (2). In contrast, our iPSC-based humanized mouse model offers a more expedited route to achieve CNS HIV-1 seeding. Our model utilizes huPBMCs that engraft rapidly within 4 weeks and efficiently in immunocompromised mice. This shortened timeline from engraftment to infection analysis distinguishes our approach from other humanized mouse models.

On the other hand, immunocompromised mice engrafted with huPBMC generate a predominantly memory and activated T-cell model. While this model supports robust and rapid acute HIV-1 infection kinetics in T-cells, it largely lacks other immune populations including peripheral monocytes and myeloid cells and naïve T-cell populations, yielding a less diverse immune system model. Umbilical cord blood and fetal tissues, rich in hematopoietic stem cells and have a high capacity for tissue regeneration, allow for more complete reconstitution of the human immune system and create a more robust humanized mouse model for studying diseases. Our iPSC-derived xenoMG system, which relies on T-cell-dependent infiltration of HIV-1 into the CNS, provides a platform for investigating HIV-1 infection using alternative approaches, such as transplantation of cord blood-derived HSC instead of huPBMC. The dual engraftment of Cord-blood HSCs and iPSC-derived MG may be a useful strategy to study the interplay between the peripheral immune system and the CNS in a humanized mouse model of HIV-1 infection.

Furthermore, our model allows for the simulation of physiological peripheral HIV-1 infection during acute human infection. During this stage, HIV-1 infects immune cells such as CD4^+^ T-cells or monocytes that traverse the blood-brain barrier, subsequently infecting microglia and disseminating HIV-1 infection in the CNS (101–106). Consequently, our humanized mouse model, dually engrafted with xenoMG and huPBMC, provides a faster alternative for achieving CNS HIV-1 seeding. It enables widespread microglial infection with proinflammatory gene expression profile activation, as demonstrated here.

Whether the infected microglia produce a virus that can disseminate extracellularly remains to be established in our system or in other humanized mouse models discussed. To date, viral outgrowth with productive infection has been demonstrated for primary microglia from a single human brain specimen, after treatment of the cultured cells with multiple chromatin-modifying drugs designed to broadly activate transcription (107).

Furthermore, our humanized mouse model also has the potential to serve as a valuable tool for quantitatively testing molecular and pharmacologic interventions. This capacity becomes especially important when assessing the impact of ART on HIV-1-infected microglia. Post-mortem brain and translational research studies have consistently demonstrated the persistence of HIV-1-infected microglia that are not eliminated by ART (15, 108–110). This persistence primarily stems from the compartmentalization of infected cells, impeding their complete eradication by ART. Therefore, it is imperative to meticulously dissect the precise kinetics and biology underlying HIV-1 immune evasion, both in the presence and absence of ART, as this ultimately leads to the formation of viral latent reservoirs and chronic HIV-1 infection. Understanding these mechanisms is critical for advancing our efforts toward developing a cure for HIV-1.

A limitation of this study is that despite confirming HIV-1 infection of microglia in the mouse brain through various methods, we have not readily detected Cre-activated GFP reporter transgene expression in the humanized mouse brain at four weeks post-infection (data not shown). Possible explanations for this may include inefficient maturation of the GFP fluorophore and/or weaker transgene expression in HIV-1-infected xenoMG. In other acute infection studies in humanized mice, the Cre-activated switch effectively marks HIV-1 DNA-positive cells for several weeks in T-cells (Satija et al. in preparation). To further develop the use of the *in vivo* Cre-activated switch, additional sequencing studies will be conducted to examine the maintenance of Cre sequence over time. The presence of the reporter transgenes in the virus may affect viral fitness potentially leading to the deletion of the transgene. While these results indicate challenges associated with using a Cre-activated reporter to track HIV-1 infection *in vivo*, further studies are needed to understand what presently limits this approach’s feasibility.

Nonetheless, our Cre-activated reporter-dependent humanized mouse model presents a powerful tool for investigating the pathogenesis of both active and latent HIV-1 infection (Satija et al. in preparation). The Cre-loxP system allows for the tracking of HIV-1-infected cells *in vivo*, enabling the characterization of viral gene expression and the identification of cellular reservoirs of latent HIV-1. In addition, transgenic reporter systems, including the one presented here, can be utilized to track human cells in the animal host. This will facilitate single-cell RNA sequencing analysis, chromatin analysis, epigenetic and molecular analysis of HIV pathogenesis, and the testing of therapeutic drug candidates against HIV-1 infection. These tools could be highly informative for examining questions related to latency and active viral expression in brain microglia before and after treatment with current, clinically approved antiretroviral drugs. Understanding the precise mechanisms of HIV-1 latency is crucial for developing strategies to eliminate latent viral reservoirs, a major barrier to an HIV-1 cure. Thus, utilizing humanized mouse models with genetically engineered iPSCs to study HIV-1 latency could provide invaluable insights into the pathogenesis of HIV-1 and the development of more effective treatments for HIV-1 infection.

## MATERIALS AND METHODS

### Construction and maintenance of fluorescent reporter iPSC

The iPSC lines were constructed at the Black Family Stem Cell Institute. The Cre recombinase-dependent dual fluorescent MSE2104 iPSC line was generated by CRISPR modification of the WTC11 line to insert a dsRed-to-eGFP cassette into the AAVS1 locus within the PPP1R12C gene (chromosomal location 19q13.4-qter) under the control of a CAG promoter. The original WTC11 line was created from PBMCs of a healthy 30-year-old male donor at the University of California San Francisco, and it has been made readily available for use by third parties for research, clinical, and commercial purposes. The MSE2104 iPSC line was maintained by culturing in feeder-free condition incomplete mTeSR E8 medium (StemCell Technologies) in a humidified incubator (5% CO_2_, 37°C) with medium changed every 1–2 days. Cells were passaged approximately every 7 days, dissociating the cells with 0.5 mM EDTA in DPBS and plated onto 6-well plates (Corning) coated with growth factor-reduced Matrigel (1 mg/mL; BD Biosciences) in mTeSR E8 medium supplemented with ROCK inhibitor Thiazovivin (Tocris). Media were switched to mTeSR E8 only medium the next day.

### iPSC differentiation into hematopoietic progenitor cells and microglia *in vitro*

HPCs and iMGs were differentiated according to a published protocol (44, 50, 51, 56). On day 0, iPSCs were passaged in mTeSR-E8 medium for a density of 20–40 colonies of 100 cells each per 35 mm well. On day 1, the cell medium was switched to STEMdiff Hematopoietic Kit (StemCell Technologies) Medium A. On day 4, the cell medium was changed to Medium B, in which the cells were differentiated into HPCs for 6–8 days. HPCs began to differentiate from the periphery of flattened endothelial cell colonies. Fully differentiated HPCs began to detach from the colonies to become suspended in medium B. On days 10–12, HPCs were harvested by collecting the medium and cells using a serological pipette. HPCs were validated by staining them with 1:100 dilution of CD34 (anti-human FITC, Biolegend), CD43 (anti-human PE, Biolegend), and CD45 (anti-human APC, BD Biosciences). Harvested HPCs were used for further differentiation *in vitro* or for HPC transplantation into neonatal mice (see below). The *in vitro* differentiation took 28–30 days in an iMG medium consisting of DMEM/F12, 2× insulin-transferrin-selenite, 2× B27, 0.5× N2, 1× glutamax, 1× non-essential amino acids, 400 mM monothioglycerol, and 5 mg/mL human insulin that was freshly supplemented with 100 ng/mL IL-34, 50 ng/mL TGFb1, and 25 ng/mL M-CSF (Peprotech). Differentiated iMGs were validated by measuring P2YR12 (anti-human PE dazzle 594, Biolegend), CD11b (anti-human APC, Biolegend), CXCR3 (anti-human BV650, Biolegend), CD45 (anti-human Alexa Fluor 700, Biolegend), CD206 (anti-human BV785, Biolegend), and CD14 (anti-human APC Fire 750, Biolegend).

### HIV-1 JRFL-Cre clone construction and cell-free HIV-1 virion production

The HIV-1 JRFL clone is a full-length molecular clone of HIV-1 based on NL4-3 (57, 88) that expresses the JRFL envelope. Cre is inserted in place of the *nef* gene, and Nef expression is restored by a downstream internal ribosome entry site (IRES) (81). Plasmid was amplified in Stbl2 electrocompetent *E. coli* and isolated using a Qiagen Midi-kit. The human epithelial 293T cell line was used to produce HIV-1 virions. 293T cells were maintained in Dulbecco’s modified Eagle medium (DMEM; Sigma) containing 10% heat-inactivated fetal bovine serum (Sigma), 100 U/mL of penicillin (Gibco), 10 U/mL of streptomycin (Gibco), and 2 mM glutamine (Gibco) (complete DMEM). Cell-free virus particles were produced by transfection of 293T cells in a 10 cm dish using Polyjet (Signajen) per the manufacturer’s protocol. Virus supernatant was harvested 48 h post-transfection, filtered with a 0.45-µm filter and concentrated by high-speed centrifugation (Sorvall ST 40R centrifuge; ThermoFisher Scientific) at 100,000*g* for 2 h at 4°C. The pelleted virus was resuspended in DPBS, aliquoted, and stored at −80°C.

### HIV-1 p24 ELISA assay

Viral stocks were quantified by NCI HIV-1 p24 ELISA kit. Corning 96-well flat-bottomed plates were coated with anti-p24 capture antibody in 0.1 M NaHCO_3_ overnight at 4°C. The plate was blocked with 1% nonfat dry milk (Lab Scientific) for 1 h. The plate was then loaded with p24 standard titrations and experimental virus supernatant treated with 1% Empigen. The dish was incubated for 2 h at room temperature or overnight at 4°C and then washed 6 times with 1× TBS-0.05% Tween (TBST). Alkaline phosphatase-conjugated mouse anti-HIV p24 (Cliniqa) was added (1:8,000 in TBST 20% sheep serum) and incubated for 1 h, followed by 6 TBST washes with TBST. The plate was developed with Sapphire Substrate (Tropix), and luminescence was quantitated on a FluoStar Optima plate reader. HIV p24 level was calculated using Prism software (GraphPad), using nonlinear standard curve regression.

### iPSC-microglia and hu-PBMC xenograft mouse model

All procedures were performed per the Institutional Animal Care and Use Committee protocol at the Icahn School of Medicine at Mount Sinai. Neonatal immunocompromised mice [C;129S4-Rag2tm1.1Flv Csf1tm1 (CSF1) Flv Il2rgtm1.1Flv/J, JAX ID# 014593] at age P0–P2 were taken out of their home cage and placed on sterile surgical drape overlying a cooling block for 2–3 min to induce hypothermic anesthesia. ICV injection of HPC was performed using a 30G needle fixed to a 10-µL Hamilton syringe. Each mouse received 400–500 K HPCs at four cranial surface coordinates at two different depths, totaling eight different sites (44). The HPCs were resuspended in 1× DPBS at 50–62.5 K cells/μL for injection. Injected mice were allowed to recover on heating pads covered with sterile surgical drapes before being returned to their home cages. Mice were weaned from their mother at P21.

At 6–10 weeks, mice hosting the central xenograft were intraperitoneally injected with human PBMCs. PBMCs were obtained from deidentified HIV-1 negative healthy blood donors (New York Blood Center), purified by Ficoll (HyClone) density gradient centrifugation, and maintained in RPMI 1640 medium (Sigma) containing 10% heat-inactivated fetal bovine serum (Sigma), 100 U/mL of penicillin (Gibco), 10 U/mL of streptomycin (Gibco), and 2 mM glutamine (Gibco) (complete RPMI). To minimize the donor variability effect, we used the same PBMC donors to inject all nine mice used for this study. PBMCs were activated with phytohemagglutinin-L (PHA-L; 2 µg/mL, Sigma) and IL-2 (50 IU/mL, Roche) for 3 days co-cultured with irradiated feeder PBMCs. Cells were harvested counted, and 10^7^ cells resuspended in 200 µL 1× PBS were intraperitoneally injected into each mouse. One week after PBMC injection, engraftment was measured weekly by quantifying human CD45^+^ cells in each mouse’s peripheral blood through fluorescence-activated cell sorting (FACS) on an Attune flow cytometer (ThermoFisher). On average, mice have successfully engrafted with PBMC ~4 weeks after the initial injection.

### PBMC engraftment FACS analysis

An Attune flow cytometer (ThermoFisher) was used to measure the level of human PBMC engraftment in our iPSC-microglia and hu-PBMC xenografted mice. The cellular layer separated from the plasma of peripheral blood was treated with ACK lysis buffer (Gibco) to remove red blood cells. Isolated white blood cells were stained with LIVE/DEAD fixable stain (Invitrogen) at a concentration of 1:1,000 in FACS buffer (2 mM EDTA, 2% FBS in DPBS) to detect live cells. Cells were incubated for 30 min at 4°C and then washed with FACS buffer. Cells were stained with 1:100 concentration of CD45 (anti-human PE-Cy7, Biolegend), CD45 (anti-mouse Pacific Blue, Biolegend), CD3 (anti-human APC eFluor780, eBiosciences), CD4 (anti-human APC, Biolegend), and CD8 (anti-human PerCP-Cy5.5, Biolegend) for 30 min at 4°C. Stained cells were washed with FACS buffer and fixed in 4% (wt/vol) PFA for FACS analysis.

### HIV-1 infection of iPSC-microglia and hu-PBMC xenografted mice

For HIV-1 infection, each mouse was injected with 250 ng HIV-1 p24 antigen either intracranially or intraperitoneally. For ICV infection, a rodent stereotaxic rig mounted with a micro pump (Stoelting) and Hamilton syringe fitted with a 30G needle was used to inject HIV-1 bilaterally into the PFC (1 µL per hemisphere) (73). The coordinates for injection were as follows: +1.5 mm anterior/posterior, ±0.5 mm medial/lateral, and 1.5 mm dorsal/ventral. The virus was injected per hemisphere at a rate of 0.25 µL per min, and four additional minutes were allowed before syringe removal. Mouse peripheral blood was collected and analyzed weekly for evidence of peripheral infection. Mice were sacrificed 4 weeks post-infection, and tissue samples were harvested and analyzed.

### Peripheral HIV-1 infection qPCR analysis

Peripheral blood collected from mice was centrifuged at 10,000*g* for 10 min to separate plasma from cells. RNA was isolated from plasma using QIAamp Viral RNA Mini kit (Qiagen) and quantified using a NanoDrop Spectrophotometer (ThermoFisher). RNA was reverse transcribed to cDNA using the High-Capacity RNA-to-cDNA kit (ThermoFisher). A Custom TaqMan Gene Expression RT-PCR assay designed for the *gag-pol* region (Assay ID: AP7DXHY; ThermoFisher) was then used on the cDNA to quantify the HIV-1 viral copy number. A series of 10-fold dilutions of measured HIV-1 target RNA fragments derived from the HIV-1 NL4-3 clone was included in each assay to generate a standard curve to derive the HIV-1 copy number.

### Immunohistochemistry of mouse brain sections and confocal microscopy

Mice were anesthetized with isoflurane and monitored for loss of consciousness. Mice that did not respond to toe pinch were cervically dislocated, and their brains dissected. Brains were drop fixed in 4% (wt/vol) PFA for 24 h. Fixed brains were cryoprotected in 30% (wt/vol) sucrose until they sank to the bottom of the solution for at least 48 h. Brains were cut coronally or sagittally at 20–40 μm thickness using a sliding microtome cooled with dry ice. Free-floating tissue sections were collected in 1× DPBS and 0.05% sodium azide. For immunohistochemistry staining, tissues were blocked in 1× DPBS, 0.1% Triton X-100, and 1% BSA for 1 h at room temperature. Tissues were incubated in primary antibodies diluted in 1× DPBS and 1% BSA overnight on a shaker at 4°C. Tissue sections were washed with DPBS three times at room temperature and incubated in fluorophore-conjugated secondary antibodies either for 1 h at room temperature or overnight at 4°C. Tissues were washed with DPBS three times at room temperature and then stained with DAPI (Sigma Aldrich). Tissues were washed with DPBS and then mounted on charged glass slides. Immunofluorescent sections were visualized and imaged using either Zeiss LSM780 or Zeiss LSM980 with airyscan2 confocal microscopes. Brightness and contrast settings were slightly adjusted for better visualization of some images. Primary antibodies: mouse anti-human nuclei (Ku80 1:100; Abcam, ab79220), mouse anti-human nuclei (HuNu 1:50; Millipore, mab1280), rabbit anti-Iba1 (1:100; Wako, 019–19741), goat anti-Iba1 (1:100; Abcam ab5076), and rabbit anti-P2ry12 (1:500; Sigma; HPA014518).

### RNAScope Fluorescence *in situ* Hybridization

Formalin-fixed and cryoprotected mouse brain tissue sections were cut into coronal and sagittal sections (10–20 µm thickness) using a freezing microtome. Sections were mounted on Superfrost Plus slides (Fisherbrand) and dried for 10 min at 60°C. The slides were processed per the RNAScope Multiplex Fluorescent v2 protocol (Advanced Cell Diagnostics). A hydrophobic barrier was drawn around the mounted sections before washing the slides with DPBS. A few drops of anti-sense probe targeting approximately 3 kb of the HIV gag-pol mRNA sequence (317691-C2, Advanced Cell Diagnostics) were added to each section and incubated for 2 h at 40°C. Slides were washed and incubated with polymerizing amplifier sequences conjugated to Opal 520 dye. Slides were washed in DPBS, briefly dried, and mounted with DAPI Fluoromount-G (Southern Biotech). Imaging was performed on a Zeiss LSM780 confocal microscope.

## Data Availability

The authors will provide all protocols and materials upon request.

## SUPPLEMENTAL MATERIAL

Figure S1 jvi.01595-23-s0001.tif MSE2104 iPSC line with a Cre-dependent dual-fluorescent reporter cassette.

Figure S2 jvi.01595-23-s0002.tif MSE2104 iPSC differentiate into hematopoietic progenitor cells (HPC) and microglia (iMG) *in vitro*.

Figure S3 jvi.01595-23-s0003.tif Quality assessment of peripheral blood T-cells.

Figure S4 jvi.01595-23-s0004.tif Two modes of HIV-1 infection tested in xenoMG mice with and without peripheral huPBMC.

Figure S5 jvi.01595-23-s0005.tif Time-course plasma HIV-1 viral load in dually xenografted humanized mice.

Supplemental legends jvi.01595-23-s0006.docx Legends for Figures S1 to S5 and Table S1.

ASM does not own the copyrights to Supplemental Material that may be linked to, or accessed through, an article. The authors have granted ASM a non-exclusive, world-wide license to publish the Supplemental Material files. Please contact the corresponding author directly for reuse.

## References

[1] World Health Organization. 2023. HIV/AIDS

[2] Honeycutt JB, Wahl A, Baker C, Spagnuolo RA, Foster J, Zakharova O, Wietgrefe S, Caro-Vegas C, Madden V, Sharpe G, Haase AT, Eron JJ, Garcia JV. 2016. Macrophages sustain HIV replication in vivo independently of T cells. J Clin Invest 126:1353–1366. doi:10.1172/JCI84456 26950420 PMC4811134

[3] Scaradavou A. 2023. Cord blood grafts for patients with HIV? Cell 186:1101–1102. doi:10.1016/j.cell.2023.01.024 36931240

[4] Siliciano JD, Kajdas J, Finzi D, Quinn TC, Chadwick K, Margolick JB, Kovacs C, Gange SJ, Siliciano RF. 2003. Long-term follow-up studies confirm the stability of the latent reservoir for HIV-1 in resting CD4^+^ T cells. Nat Med 9:727–728. doi:10.1038/nm880 12754504

[5] Lee E, von Stockenstrom S, Morcilla V, Odevall L, Hiener B, Shao W, Hartogensis W, Bacchetti P, Milush J, Liegler T, Sinclair E, Hatano H, Hoh R, Somsouk M, Hunt P, Boritz E, Douek D, Fromentin R, Chomont N, Deeks SG, Hecht FM, Palmer S. 2020. Impact of antiretroviral therapy duration on HIV-1 infection of T cells within anatomic sites. J Virol 94:e01270-19. doi:10.1128/JVI.01270-19 31723024 PMC7000983

[6] Chun T-W, Justement JS, Murray D, Hallahan CW, Maenza J, Collier AC, Sheth PM, Kaul R, Ostrowski M, Moir S, Kovacs C, Fauci AS. 2010. Rebound of plasma viremia following cessation of antiretroviral therapy despite profoundly low levels of HIV reservoir: implications for eradication. AIDS 24:2803–2808. doi:10.1097/QAD.0b013e328340a239 20962613 PMC3154092

[7] Cho A, Gaebler C, Olveira T, Ramos V, Saad M, Lorenzi JCC, Gazumyan A, Moir S, Caskey M, Chun TW, Nussenzweig MC. 2022. Longitudinal clonal dynamics of HIV-1 latent reservoirs measured by combination quadruplex polymerase chain reaction and sequencing. Proc Natl Acad Sci U S A 119:e2117630119. doi:10.1073/pnas.2117630119 35042816 PMC8794825

[8] Collora JA, Ho Y-C. 2022. The loud minority: transcriptionally active HIV-1-infected cells survive, proliferate, and persist. Cell 185:227–229. doi:10.1016/j.cell.2021.12.038 35063069 PMC9195179

[9] White JA, Kufera JT, Bachmann N, Dai W, Simonetti FR, Armstrong C, Lai J, Beg S, Siliciano JD, Siliciano RF. 2022. Measuring the latent reservoir for HIV-1: quantification bias in near full-length genome sequencing methods. PLoS Pathog 18:e1010845. doi:10.1371/journal.ppat.1010845 36074794 PMC9488763

[10] Nasi M, De Biasi S, Gibellini L, Bianchini E, Pecorini S, Bacca V, Guaraldi G, Mussini C, Pinti M, Cossarizza A. 2017. Ageing and inflammation in patients with HIV infection. Clin Exp Immunol 187:44–52. doi:10.1111/cei.12814 27198731 PMC5167025

[11] Sieg SF, Shive CL, Panigrahi S, Freeman ML. 2021. Probing the interface of HIV and inflammaging. Curr HIV/AIDS Rep 18:198–210. doi:10.1007/s11904-021-00547-0 33709322

[12] Kaplan-Lewis E, Aberg JA, Lee M. 2017. Aging with HIV in the ART era. Semin Diagn Pathol 34:384–397. doi:10.1053/j.semdp.2017.04.002 28552209

[13] Deeks SG. 2011. HIV infection, inflammation, immunosenescence, and aging. Annu Rev Med 62:141–155. doi:10.1146/annurev-med-042909-093756 21090961 PMC3759035

[14] Guo M-L, Buch S. 2019. Neuroinflammation & pre-mature aging in the context of chronic HIV infection and drug abuse: role of dysregulated autophagy. Brain Res 1724:146446. doi:10.1016/j.brainres.2019.146446 31521638 PMC6933726

[15] Plaza-Jennings AL, Valada A, O’Shea C, Iskhakova M, Hu B, Javidfar B, Ben Hutta G, Lambert TY, Murray J, Kassim B, Chandrasekaran S, Chen BK, Morgello S, Won H, Akbarian S. 2022. HIV integration in the human brain is linked to microglial activation and 3D genome remodeling. Mol Cell 82:4647–4663. doi:10.1016/j.molcel.2022.11.016 36525955 PMC9831062

[16] Heaton RK, Clifford DB, Franklin DR, Woods SP, Ake C, Vaida F, Ellis RJ, Letendre SL, Marcotte TD, Atkinson JH, et al.. 2010. HIV-associated neurocognitive disorders persist in the era of potent antiretroviral therapy: CHARTER Study. Neurology 75:2087–2096. doi:10.1212/WNL.0b013e318200d727 21135382 PMC2995535

[17] Heaton RK, Franklin DR, Ellis RJ, McCutchan JA, Letendre SL, Leblanc S, Corkran SH, Duarte NA, Clifford DB, Woods SP, et al.. 2011. HIV-associated neurocognitive disorders before and during the era of combination antiretroviral therapy: differences in rates, nature, and predictors. J Neurovirol 17:3–16. doi:10.1007/s13365-010-0006-1 21174240 PMC3032197

[18] Eggers C, Arendt G, Hahn K, Husstedt IW, Maschke M, Neuen-Jacob E, Obermann M, Rosenkranz T, Schielke E, Straube E, German Association of Neuro-AIDS und Neuro-Infectiology (DGNANI). 2017. HIV-1-associated neurocognitive disorder: epidemiology, pathogenesis, diagnosis, and treatment. J Neurol 264:1715–1727. doi:10.1007/s00415-017-8503-2 28567537 PMC5533849

[19] Kahn JO, Walker BD. 1998. Acute human immunodeficiency virus type 1 infection. N Engl J Med 339:33–39. doi:10.1056/NEJM199807023390107 9647878

[20] Davis LE, Hjelle BL, Miller VE, Palmer DL, Llewellyn AL, Merlin TL, Young SA, Mills RG, Wachsman W, Wiley CA. 1992. Early viral brain invasion in iatrogenic human immunodeficiency virus infection. Neurology 42:1736–1739. doi:10.1212/wnl.42.9.1736 1513462

[21] Longino AA, Paul R, Wang Y, Lama JR, Brandes P, Ruiz E, Correa C, Keating S, Spudich SS, Pilcher C, Vecchio A, Pasalar S, Bender Ignacio RA, Valdez R, Dasgupta S, Robertson K, Duerr A. 2022. HIV disease dynamics and markers of inflammation and CNS injury during primary HIV infection and their relationship to cognitive performance. J Acquir Immune Defic Syndr 89:183–190. doi:10.1097/QAI.0000000000002832 34629415 PMC8752485

[22] Valcour V, Chalermchai T, Sailasuta N, Marovich M, Lerdlum S, Suttichom D, Suwanwela NC, Jagodzinski L, Michael N, Spudich S, van Griensven F, de Souza M, Kim J, Ananworanich J, RV254/SEARCH 010 Study Group. 2012. Central nervous system viral invasion and inflammation during acute HIV infection. J Infect Dis 206:275–282. doi:10.1093/infdis/jis326 22551810 PMC3490695

[23] León-Rivera R, Veenstra M, Donoso M, Tell E, Eugenin EA, Morgello S, Berman JW. 2021. Central nervous system (CNS) viral seeding by mature monocytes and potential therapies to reduce CNS viral reservoirs in the cART era. mBio 12:e03633-20. doi:10.1128/mBio.03633-20 33727362 PMC8092320

[24] León-Rivera R, Morsey B, Niu M, Fox HS, Berman JW. 2020. Interactions of monocytes, HIV, and ART identified by an innovative scRNAseq pipeline: pathways to reservoirs and HIV-associated comorbidities. mBio 11:e01037-20. doi:10.1128/mBio.01037-20 32723919 PMC7387797

[25] Veenstra M, Leon-Rivera R, Li M, Gama L, Clements JE, Berman JW. 2017. Mechanisms of CNS viral seeding by HIV^+^ CD14^+^ CD16^+^ monocytes: establishment and reseeding of viral reservoirs contributing to HIV-associated neurocognitive disorders. mBio 8:e01280-17. doi:10.1128/mbio.01280-17 29066542 PMC5654927

[26] Hong S, Banks WA. 2015. Role of the immune system in HIV-associated neuroinflammation and neurocognitive implications. Brain Behav Immun 45:1–12. doi:10.1016/j.bbi.2014.10.008 25449672 PMC4342286

[27] Ho DD, Rota TR, Hirsch MS. 1986. Infection of monocyte/macrophages by human T lymphotropic virus type III. J Clin Invest 77:1712–1715. doi:10.1172/JCI112491 2422213 PMC424579

[28] Li H, McLaurin KA, Illenberger JM, Mactutus CF, Booze RM. 2021. Microglial HIV-1 expression: role in HIV-1 associated neurocognitive disorders. Viruses 13:924. doi:10.3390/v13050924 34067600 PMC8155894

[29] Li Q, Barres BA. 2018. Microglia and macrophages in brain homeostasis and disease. Nat Rev Immunol 18:225–242. doi:10.1038/nri.2017.125 29151590

[30] von Herrath M, Oldstone MB, Fox HS. 1995. Simian immunodeficiency virus (SIV)-specific CTL in cerebrospinal fluid and brains of SIV-infected rhesus macaques. J Immunol 154:5582–5589. doi:10.4049/jimmunol.154.10.5582 7730657

[31] Byrnes SJ, Angelovich TA, Busman-Sahay K, Cochrane CR, Roche M, Estes JD, Churchill MJ. 2022. Non-human primate models of HIV brain infection and cognitive disorders. Viruses 14:1997. doi:10.3390/v14091997 36146803 PMC9500831

[32] Hatziioannou T, Evans DT. 2012. Animal models for HIV/AIDS research. Nat Rev Microbiol 10:852–867. doi:10.1038/nrmicro2911 23154262 PMC4334372

[33] Potash MJ, Chao W, Bentsman G, Paris N, Saini M, Nitkiewicz J, Belem P, Sharer L, Brooks AI, Volsky DJ. 2005. A mouse model for study of systemic HIV-1 infection, antiviral immune responses, and neuroinvasiveness. Proc Natl Acad Sci U S A 102:3760–3765. doi:10.1073/pnas.0500649102 15728729 PMC553332

[34] Gillgrass A, Wessels JM, Yang JX, Kaushic C. 2020. Advances in humanized mouse models to improve understanding of HIV-1 pathogenesis and immune responses. Front Immunol 11:617516. doi:10.3389/fimmu.2020.617516 33746940 PMC7973037

[35] Flerin NC, Bardhi A, Zheng JH, Korom M, Folkvord J, Kovacs C, Benko E, Truong R, Mota T, Connick E, Jones RB, Lynch RM, Goldstein H. 2019. Establishment of a novel humanized mouse model to investigate in vivo activation and depletion of patient-derived HIV latent reservoirs. J Virol 93:e02051-18. doi:10.1128/JVI.02051-18 30626677 PMC6401459

[36] Mathews S, Branch Woods A, Katano I, Makarov E, Thomas MB, Gendelman HE, Poluektova LY, Ito M, Gorantla S. 2019. Human interleukin-34 facilitates microglia-like cell differentiation and persistent HIV-1 infection in humanized mice. Mol Neurodegener 14:12. doi:10.1186/s13024-019-0311-y 30832693 PMC6399898

[37] Llewellyn GN, Alvarez-Carbonell D, Chateau M, Karn J, Cannon PM. 2018. HIV-1 infection of microglial cells in a reconstituted humanized mouse model and identification of compounds that selectively reverse HIV latency. J Neurovirol 24:192–203. doi:10.1007/s13365-017-0604-2 29256041 PMC5910454

[38] Zhang J, Lohani SC, Cheng Y, Wang T, Guo L, Kim WK, Gorantla S, Li Q. 2021. Human microglia extensively reconstitute in humanized-BLT mice with human interleukin-34 transgene and support HIV-1 brain infection. Front Immunol 12:672415. doi:10.3389/fimmu.2021.672415 34093573 PMC8176960

[39] Gavegnano C, Haile W, Koneru R, Hurwitz SJ, Kohler JJ, Tyor WR, Schinazi RF. 2020. Novel method to quantify phenotypic markers of HIV-associated neurocognitive disorder in a murine SCID model. J Neurovirol 26:838–845. doi:10.1007/s13365-020-00842-3 32901392 PMC7718289

[40] Hiwarkar P, Hubank M, Qasim W, Chiesa R, Gilmour KC, Saudemont A, Amrolia PJ, Veys P. 2017. Cord blood transplantation recapitulates fetal ontogeny with a distinct molecular signature that supports CD4^+^ T-cell reconstitution. Blood Adv 1:2206–2216. doi:10.1182/bloodadvances.2017010827 29296868 PMC5737134

[41] Honeycutt JB, Sheridan PA, Matsushima GK, Garcia JV. 2015. Humanized mouse models for HIV-1 infection of the CNS. J Neurovirol 21:301–309. doi:10.1007/s13365-014-0299-6 25366661 PMC4418936

[42] Kreitzer FR, Salomonis N, Sheehan A, Huang M, Park JS, Spindler MJ, Lizarraga P, Weiss WA, So P-L, Conklin BR. 2013. A robust method to derive functional neural crest cells from human pluripotent stem cells. Am J Stem Cells 2:119–131. 23862100 PMC3708511

[43] Roberts B, Haupt A, Tucker A, Grancharova T, Arakaki J, Fuqua MA, Nelson A, Hookway C, Ludmann SA, Mueller IA, Yang R, Horwitz R, Rafelski SM, Gunawardane RN. 2017. Systematic gene tagging using CRISPR/Cas9 in human stem cells to illuminate cell organization. Mol Biol Cell 28:2854–2874. doi:10.1091/mbc.E17-03-0209 28814507 PMC5638588

[44] Hasselmann J, Coburn MA, England W, Figueroa Velez DX, Kiani Shabestari S, Tu CH, McQuade A, Kolahdouzan M, Echeverria K, Claes C, Nakayama T, Azevedo R, Coufal NG, Han CZ, Cummings BJ, Davtyan H, Glass CK, Healy LM, Gandhi SP, Spitale RC, Blurton-Jones M. 2019. Development of a chimeric model to study and manipulate human microglia in vivo. Neuron 103:1016–1033. doi:10.1016/j.neuron.2019.07.002 31375314 PMC7138101

[45] Dräger NM, Sattler SM, Huang C-L, Teter OM, Leng K, Hashemi SH, Hong J, Aviles G, Clelland CD, Zhan L, Udeochu JC, Kodama L, Singleton AB, Nalls MA, Ichida J, Ward ME, Faghri F, Gan L, Kampmann M. 2022. A CRISPRi/a platform in human iPSC-derived microglia uncovers regulators of disease states. Nat Neurosci 25:1149–1162. doi:10.1038/s41593-022-01131-4 35953545 PMC9448678

[46] Irion S, Luche H, Gadue P, Fehling HJ, Kennedy M, Keller G. 2007. Identification and targeting of the ROSA26 locus in human embryonic stem cells. Nat Biotechnol 25:1477–1482. doi:10.1038/nbt1362 18037879

[47] Lombardo A, Cesana D, Genovese P, Di Stefano B, Provasi E, Colombo DF, Neri M, Magnani Z, Cantore A, Lo Riso P, Damo M, Pello OM, Holmes MC, Gregory PD, Gritti A, Broccoli V, Bonini C, Naldini L. 2011. Site-specific integration and tailoring of cassette design for sustainable gene transfer. Nat Methods 8:861–869. doi:10.1038/nmeth.1674 21857672

[48] Macarthur CC, Xue H, Van Hoof D, Lieu PT, Dudas M, Fontes A, Swistowski A, Touboul T, Seerke R, Laurent LC, Loring JF, German MS, Zeng X, Rao MS, Lakshmipathy U, Chesnut JD, Liu Y. 2012. Chromatin insulator elements block transgene silencing in engineered human embryonic stem cell lines at a defined chromosome 13 locus. Stem Cells Dev 21:191–205. doi:10.1089/scd.2011.0163 21699412 PMC3258440

[49] Oceguera-Yanez F, Kim S-I, Matsumoto T, Tan GW, Xiang L, Hatani T, Kondo T, Ikeya M, Yoshida Y, Inoue H, Woltjen K. 2016. Engineering the AAVS1 locus for consistent and scalable transgene expression in human iPSCs and their differentiated derivatives. Methods 101:43–55. doi:10.1016/j.ymeth.2015.12.012 26707206

[50] Abud EM, Ramirez RN, Martinez ES, Healy LM, Nguyen CHH, Newman SA, Yeromin AV, Scarfone VM, Marsh SE, Fimbres C, Caraway CA, Fote GM, Madany AM, Agrawal A, Kayed R, Gylys KH, Cahalan MD, Cummings BJ, Antel JP, Mortazavi A, Carson MJ, Poon WW, Blurton-Jones M. 2017. iPSC-derived human microglia-like cells to study neurological diseases. Neuron 94:278–293. doi:10.1016/j.neuron.2017.03.042 28426964 PMC5482419

[51] McQuade A, Coburn M, Tu CH, Hasselmann J, Davtyan H, Blurton-Jones M. 2018. Development and validation of a simplified method to generate human microglia from pluripotent stem cells. Mol Neurodegener 13:67. doi:10.1186/s13024-018-0297-x 30577865 PMC6303871

[52] Svoboda DS, Barrasa MI, Shu J, Rietjens R, Zhang S, Mitalipova M, Berube P, Fu D, Shultz LD, Bell GW, Jaenisch R. 2019. Human iPSC-derived microglia assume a primary microglia-like state after transplantation into the neonatal mouse brain. Proc Natl Acad Sci U S A 116:25293–25303. doi:10.1073/pnas.1913541116 31772018 PMC6911218

[53] Xu R, Li X, Boreland AJ, Posyton A, Kwan K, Hart RP, Jiang P. 2020. Human iPSC-derived mature microglia retain their identity and functionally integrate in the chimeric mouse brain. Nat Commun 11:1577. doi:10.1038/s41467-020-15411-9 32221280 PMC7101330

[54] Bozhilov YK, Hsu I, Brown EJ, Wilkinson AC. 2023. In vitro human haematopoietic stem cell expansion and differentiation. Cells 12:896. doi:10.3390/cells12060896 36980237 PMC10046976

[55] Choi K-D, Vodyanik M, Slukvin II. 2011. Hematopoietic differentiation and production of mature myeloid cells from human pluripotent stem cells. Nat Protoc 6:296–313. doi:10.1038/nprot.2010.184 21372811 PMC3066067

[56] McQuade A, Blurton-Jones M. 2022. Human induced pluripotent stem cell-derived microglia (hiPSC-Microglia). Methods Mol Biol 2454:473–482. doi:10.1007/7651_2021_429 34773245

[57] O’Brien WA, Koyanagi Y, Namazie A, Zhao JQ, Diagne A, Idler K, Zack JA, Chen IS. 1990. HIV-1 tropism for mononuclear phagocytes can be determined by regions of gp120 outside the CD4-binding domain. Nature 348:69–73. doi:10.1038/348069a0 2172833

[58] Costantino CM, Gupta A, Yewdall AW, Dale BM, Devi LA, Chen BK. 2012. Cannabinoid receptor 2-mediated attenuation of CXCR4-tropic HIV infection in primary CD4^+^ T cells. PLoS One 7:e33961. doi:10.1371/journal.pone.0033961 22448282 PMC3309010

[59] Durham ND, Chen BK. 2016. Measuring T cell-to-T cell HIV-1 transfer, viral fusion, and infection using flow cytometry. Methods Mol Biol 1354:21–38. doi:10.1007/978-1-4939-3046-3_2 26714702 PMC6295342

[60] Ghorpade A, Nukuna A, Che M, Haggerty S, Persidsky Y, Carter E, Carhart L, Shafer L, Gendelman HE. 1998. Human immunodeficiency virus neurotropism: an analysis of viral replication and cytopathicity for divergent strains in monocytes and microglia. J Virol 72:3340–3350. doi:10.1128/JVI.72.4.3340-3350.1998 9525661 PMC109814

[61] Lee SC, Hatch WC, Liu W, Kress Y, Lyman WD, Dickson DW. 1993. Productive infection of human fetal microglia by HIV-1. Am J Pathol 143:1032–1039. 8213999 PMC1887063

[62] Sharpless N, Gilbert D, Vandercam B, Zhou JM, Verdin E, Ronnett G, Friedman E, Dubois-Dalcq M. 1992. The restricted nature of HIV-1 tropism for cultured neural cells. Virology 191:813–825. doi:10.1016/0042-6822(92)90257-p 1448925

[63] Broder CC, Berger EA. 1995. Fusogenic selectivity of the envelope glycoprotein is a major determinant of human immunodeficiency virus type 1 tropism for CD4^+^ T-cell lines vs. primary macrophages. Proc Natl Acad Sci U S A 92:9004–9008. doi:10.1073/pnas.92.19.9004 7568061 PMC41096

[64] Esposito AM, Cheung P, Swartz TH, Li H, Tsibane T, Durham ND, Basler CF, Felsenfeld DP, Chen BK. 2016. A high throughput Cre-lox activated viral membrane fusion assay identifies pharmacological inhibitors of HIV entry. Virology 490:6–16. doi:10.1016/j.virol.2015.10.013 26803470 PMC5019951

[65] Chen BK, Gandhi RT, Baltimore D. 1996. CD4 down-modulation during infection of human T cells with human immunodeficiency virus type 1 involves independent activities of vpu, env, and nef. J Virol 70:6044–6053. doi:10.1128/JVI.70.9.6044-6053.1996 8709227 PMC190625

[66] Law KM, Komarova NL, Yewdall AW, Lee RK, Herrera OL, Wodarz D, Chen BK. 2016. In vivo HIV-1 cell-to-cell transmission promotes multicopy micro-compartmentalized infection. Cell Rep 15:2771–2783. doi:10.1016/j.celrep.2016.05.059 27292632

[67] Durham ND, Chen BK. 2015. HIV-1 cell-free and cell-to-cell infections are differentially regulated by distinct determinants in the Env gp41 cytoplasmic tail. J Virol 89:9324–9337. doi:10.1128/JVI.00655-15 26136566 PMC4542346

[68] Verkhusha VV, Kuznetsova IM, Stepanenko OV, Zaraisky AG, Shavlovsky MM, Turoverov KK, Uversky VN. 2003. High stability of Discosoma DsRed as compared to Aequorea EGFP. Biochemistry 42:7879–7884. doi:10.1021/bi034555t 12834339

[69] Baird GS, Zacharias DA, Tsien RY. 2000. Biochemistry, mutagenesis, and oligomerization of DsRed, a red fluorescent protein from coral. Proc Natl Acad Sci U S A 97:11984–11989. doi:10.1073/pnas.97.22.11984 11050229 PMC17281

[70] Jin M, Ma Z, Jiang P. 2022. Generation of iPSC-based human-mouse microglial brain chimeras to study senescence of human microglia. STAR Protoc 3:101847. doi:10.1016/j.xpro.2022.101847 36595906 PMC9667309

[71] Kim KC, Choi B-S, Kim K-C, Park KH, Lee HJ, Cho YK, Kim SI, Kim SS, Oh Y-K, Kim YB. 2016. A simple mouse model for the study of human immunodeficiency virus. AIDS Res Hum Retroviruses 32:194–202. doi:10.1089/AID.2015.0211 26564392 PMC4761813

[72] Dash PK, Gorantla S, Poluektova L, Hasan M, Waight E, Zhang C, Markovic M, Edagwa B, Machhi J, Olson KE, Wang X, Mosley RL, Kevadiya B, Gendelman HE. 2021. Humanized mice for infectious and neurodegenerative disorders. Retrovirology 18:13. doi:10.1186/s12977-021-00557-1 34090462 PMC8179712

[73] Mitchell AC, Javidfar B, Bicks LK, Neve R, Garbett K, Lander SS, Mirnics K, Morishita H, Wood MA, Jiang Y, Gaisler-Salomon I, Akbarian S. 2016. Longitudinal assessment of neuronal 3D genomes in mouse prefrontal cortex. Nat Commun 7:12743. doi:10.1038/ncomms12743 27597321 PMC5025847

[74] Vlahov D, Graham N, Hoover D, Flynn C, Bartlett JG, Margolick JB, Lyles CM, Nelson KE, Smith D, Holmberg S, Farzadegan H. 1998. Prognostic indicators for AIDS and infectious disease death in HIV-infected injection drug users: plasma viral load and CD4^+^ cell count. JAMA 279:35–40. doi:10.1001/jama.279.1.35 9424041

[75] Mellors JW, Rinaldo Jr CR, Gupta P, White RM, Todd JA, Kingsley LA. 1996. Prognosis in HIV-1 infection predicted by the quantity of virus in plasma. Science 272:1167–1170. doi:10.1126/science.272.5265.1167 8638160

[76] Mellors JW, Muñoz A, Giorgi JV, Margolick JB, Tassoni CJ, Gupta P, Kingsley LA, Todd JA, Saah AJ, Detels R, Phair JP, Rinaldo Jr CR. 1997. Plasma viral load and CD4^+^ lymphocytes as prognostic markers of HIV-1 infection. Ann Intern Med 126:946–954. doi:10.7326/0003-4819-126-12-199706150-00003 9182471

[77] Morgello S. 2018. HIV neuropathology. Handb Clin Neurol 152:3–19. doi:10.1016/B978-0-444-63849-6.00002-5 29604982

[78] Solomon IH, Chettimada S, Misra V, Lorenz DR, Gorelick RJ, Gelman BB, Morgello S, Gabuzda D. 2020. White matter abnormalities linked to interferon, stress response, and energy metabolism gene expression changes in older HIV-positive patients on antiretroviral therapy. Mol Neurobiol 57:1115–1130. doi:10.1007/s12035-019-01795-3 31691183 PMC7035207

[79] Davalos D, Grutzendler J, Yang G, Kim JV, Zuo Y, Jung S, Littman DR, Dustin ML, Gan W-B. 2005. ATP mediates rapid microglial response to local brain injury in vivo. Nat Neurosci 8:752–758. doi:10.1038/nn1472 15895084

[80] Wolf SA, Boddeke H, Kettenmann H. 2017. Microglia in physiology and disease. Annu Rev Physiol 79:619–643. doi:10.1146/annurev-physiol-022516-034406 27959620

[81] Brabers N, Nottet H. 2006. Role of the pro-inflammatory cytokines TNF-alpha and IL-1beta in HIV-associated dementia. Eur J Clin Invest 36:447–458. doi:10.1111/j.1365-2362.2006.01657.x 16796601

[82] Dubrovsky L, Brichacek B, Prashant NM, Pushkarsky T, Mukhamedova N, Fleetwood AJ, Xu Y, Dragoljevic D, Fitzgerald M, Horvath A, Murphy AJ, Sviridov D, Bukrinsky MI. 2022. Extracellular vesicles carrying HIV-1 Nef induce long-term hyperreactivity of myeloid cells. Cell Rep 41:111674. doi:10.1016/j.celrep.2022.111674 36417867 PMC9733434

[83] Wahl A, Al-Harthi L. 2023. HIV infection of non-classical cells in the brain. Retrovirology 20:1. doi:10.1186/s12977-023-00616-9 36639783 PMC9840342

[84] D’Souza SS, Maufort J, Kumar A, Zhang J, Smuga-Otto K, Thomson JA, Slukvin II. 2016. GSK3β inhibition promotes efficient myeloid and lymphoid hematopoiesis from non-human primate-induced pluripotent stem cells. Stem Cell Reports 6:243–256. doi:10.1016/j.stemcr.2015.12.010 26805448 PMC4750098

[85] Ryan SK, Gonzalez MV, Garifallou JP, Bennett FC, Williams KS, Sotuyo NP, Mironets E, Cook K, Hakonarson H, Anderson SA, Jordan-Sciutto KL. 2020. Neuroinflammation and EIF2 signaling persist despite antiretroviral treatment in an hiPSC tri-culture model of HIV infection. Stem Cell Reports 14:991. doi:10.1016/j.stemcr.2020.04.006 32402270 PMC7221088

[86] Eltalkhawy YM, Takahashi N, Ariumi Y, Shimizu J, Miyazaki K, Senju S, Suzu S. 2023. iPS cell-derived model to study the interaction between tissue macrophage and HIV-1. J Leukoc Biol 114:53–67. doi:10.1093/jleuko/qiad024 36976024

[87] Li G-H, Maric D, Major EO, Nath A. 2020. Productive HIV infection in astrocytes can be established via a nonclassical mechanism. AIDS 34:963–978. doi:10.1097/QAD.0000000000002512 32379159 PMC7429268

[88] Allers K, Hütter G, Hofmann J, Loddenkemper C, Rieger K, Thiel E, Schneider T. 2011. Evidence for the cure of HIV infection by CCR5Δ32/Δ32 stem cell transplantation. Blood 117:2791–2799. doi:10.1182/blood-2010-09-309591 21148083

[89] Gupta RK, Peppa D, Hill AL, Gálvez C, Salgado M, Pace M, McCoy LE, Griffith SA, Thornhill J, Alrubayyi A, Huyveneers LEP, Nastouli E, Grant P, Edwards SG, Innes AJ, Frater J, Nijhuis M, Wensing AMJ, Martinez-Picado J, Olavarria E. 2020. Evidence for HIV-1 cure after CCR5Δ32/Δ32 allogeneic haemopoietic stem-cell transplantation 30 months post analytical treatment interruption: a case report. Lancet HIV 7:e340–e347. doi:10.1016/S2352-3018(20)30069-2 32169158 PMC7606918

[90] Gupta RK, Abdul-Jawad S, McCoy LE, Mok HP, Peppa D, Salgado M, Martinez-Picado J, Nijhuis M, Wensing AMJ, Lee H, Grant P, Nastouli E, Lambert J, Pace M, Salasc F, Monit C, Innes AJ, Muir L, Waters L, Frater J, Lever AML, Edwards SG, Gabriel IH, Olavarria E. 2019. HIV-1 remission following CCR5Δ32/Δ32 haematopoietic stem-cell transplantation. Nature 568:244–248. doi:10.1038/s41586-019-1027-4 30836379 PMC7275870

[91] Anonymous. 2023. HIV-1 cure after CCR5Δ32/Δ32 allogeneic hematopoietic stem cell transplantation. Nat Med 29:547–548. doi:10.1038/s41591-023-02215-9 36849733

[92] Hsu J, Van Besien K, Glesby MJ, Pahwa S, Coletti A, Warshaw MG, Petz LD, Moore TB, Chen YH, Pallikkuth S, et al.. 2023. HIV-1 remission and possible cure in a woman after haplo-cord blood transplant. Cell 186:1115–1126. doi:10.1016/j.cell.2023.02.030 36931242 PMC10616809

[93] Behrendt R, Schumann T, Gerbaulet A, Nguyen LA, Schubert N, Alexopoulou D, Berka U, Lienenklaus S, Peschke K, Gibbert K, Wittmann S, Lindemann D, Weiss S, Dahl A, Naumann R, Dittmer U, Kim B, Mueller W, Gramberg T, Roers A. 2013. Mouse SAMHD1 has antiretroviral activity and suppresses a spontaneous cell-intrinsic antiviral response. Cell Rep 4:689–696. doi:10.1016/j.celrep.2013.07.037 23972988 PMC4807655

[94] Rehwinkel J, Maelfait J, Bridgeman A, Rigby R, Hayward B, Liberatore RA, Bieniasz PD, Towers GJ, Moita LF, Crow YJ, Bonthron DT, Reis e Sousa C. 2013. SAMHD1-dependent retroviral control and escape in mice. EMBO J 32:2454–2462. doi:10.1038/emboj.2013.163 23872947 PMC3770946

[95] Lahouassa H, Daddacha W, Hofmann H, Ayinde D, Logue EC, Dragin L, Bloch N, Maudet C, Bertrand M, Gramberg T, Pancino G, Priet S, Canard B, Laguette N, Benkirane M, Transy C, Landau NR, Kim B, Margottin-Goguet F. 2012. SAMHD1 restricts the replication of human immunodeficiency virus type 1 by depleting the intracellular pool of deoxynucleoside triphosphates. Nat Immunol 13:223–228. doi:10.1038/ni.2236 22327569 PMC3771401

[96] Bloch N, Gläsker S, Sitaram P, Hofmann H, Shepard CN, Schultz ML, Kim B, Landau NR. 2017. A highly active isoform of lentivirus restriction factor SAMHD1 in mouse. J Biol Chem 292:1068–1080. doi:10.1074/jbc.M116.743740 27920203 PMC5247641

[97] Kim B, Nguyen LA, Daddacha W, Hollenbaugh JA. 2012. Tight interplay among SAMHD1 protein level, cellular dNTP levels, and HIV-1 proviral DNA synthesis kinetics in human primary monocyte-derived macrophages. J Biol Chem 287:21570–21574. doi:10.1074/jbc.C112.374843 22589553 PMC3381122

[98] Honeycutt JB, Garcia JV. 2018. Humanized mice: models for evaluating NeuroHIV and cure strategies. J Neurovirol 24:185–191. doi:10.1007/s13365-017-0567-3 28831774 PMC6506160

[99] Waight E, Zhang C, Mathews S, Kevadiya BD, Lloyd KCK, Gendelman HE, Gorantla S, Poluektova LY, Dash PK. 2022. Animal models for studies of HIV-1 brain reservoirs. J Leukoc Biol 112:1285–1295. doi:10.1002/JLB.5VMR0322-161R 36044375 PMC9804185

[100] Wu X, Liu L, Cheung K-W, Wang H, Lu X, Cheung AKL, Liu W, Huang X, Li Y, Chen ZW, Chen SMY, Zhang T, Wu H, Chen Z. 2016. Brain invasion by CD4^+^ T cells infected with a transmitted/founder HIV-1_BJZS7_ during acute stage in humanized mice. J Neuroimmune Pharmacol 11:572–583. doi:10.1007/s11481-016-9654-0 26838362

[101] Kincer LP, Schnell G, Swanstrom R, Miller MB, Spudich S, Eron JJ, Price RW, Joseph SB. 2022. HIV-1 is transported into the central nervous system by trafficking infected cells. Pathog Immun 7:131–142. doi:10.20411/pai.v7i2.524 36865569 PMC9973728

[102] Rahimy E, Li F-Y, Hagberg L, Fuchs D, Robertson K, Meyerhoff DJ, Zetterberg H, Price RW, Gisslén M, Spudich S. 2017. Blood-brain barrier disruption is initiated during primary HIV infection and not rapidly altered by antiretroviral therapy. J Infect Dis 215:1132–1140. doi:10.1093/infdis/jix013 28368497 PMC5426376

[103] Spudich S, González-Scarano F. 2012. HIV-1-related central nervous system disease: current issues in pathogenesis, diagnosis, and treatment. Cold Spring Harb Perspect Med 2:a007120. doi:10.1101/cshperspect.a007120 22675662 PMC3367536

[104] Osborne O, Peyravian N, Nair M, Daunert S, Toborek M. 2020. The paradox of HIV blood-brain barrier penetrance and antiretroviral drug delivery deficiencies. Trends Neurosci 43:695–708. doi:10.1016/j.tins.2020.06.007 32682564 PMC7483662

[105] Haase AT. 1986. Pathogenesis of lentivirus infections. Nature 322:130–136. doi:10.1038/322130a0 2425264

[106] Peluso R, Haase A, Stowring L, Edwards M, Ventura P. 1985. A Trojan Horse mechanism for the spread of visna virus in monocytes. Virology 147:231–236. doi:10.1016/0042-6822(85)90246-6 2998068

[107] Tang Y, Chaillon A, Gianella S, Wong LM, Li D, Simermeyer TL, Porrachia M, Ignacio C, Woodworth B, Zhong D, et al.. 2023. Brain microglia serve as a persistent HIV reservoir despite durable antiretroviral therapy. J Clin Invest 133:e167417. doi:10.1172/JCI167417 37317962 PMC10266791

[108] Jones BR, Miller RL, Kinloch NN, Tsai O, Rigsby H, Sudderuddin H, Shahid A, Ganase B, Brumme CJ, Harris M, Poon AFY, Brockman MA, Fromentin R, Chomont N, Joy JB, Brumme ZL. 2020. Genetic diversity, compartmentalization, and age of HIV poviruses persisting in CD4^+^ T cell subsets during long-term combination antiretroviral therapy. J Virol 94:e01786-19. doi:10.1128/JVI.01786-19 31776273 PMC7022348

[109] Omondi FH, Sudderuddin H, Shahid A, Kinloch NN, Jones BR, Miller RL, Tsai O, MacMillan D, Trocha A, Brockman MA, Brumme CJ, Joy JB, Liang R, Walker BD, Brumme ZL. 2021. HIV proviral burden, genetic diversity, and dynamics in viremic controllers who subsequently initiated suppressive antiretroviral therapy. mBio 12:e0249021. doi:10.1128/mBio.02490-21 34781741 PMC8693448

[110] Sun W, Rassadkina Y, Gao C, Collens SI, Lian X, Solomon IH, Mukerji S, Yu XG, Lichterfeld M. 2023. Persistence of intact HIV-1 proviruses in the brain during antiretroviral therapy. bioRxiv:2023.06.26.546135. doi:10.1101/2023.06.26.546135 PMC10631759 37938115

