# Supplementary figures and images for: HIV-1 infection of genetically engineered iPSC-derived central nervous system-engrafted microglia in a humanized mouse model
Source: J Virol. 2023 Nov 30;97(12):e01595-23. doi: 10.1128/jvi.01595-23 (PMC10734545; doi:10.1128/jvi.01595-23)

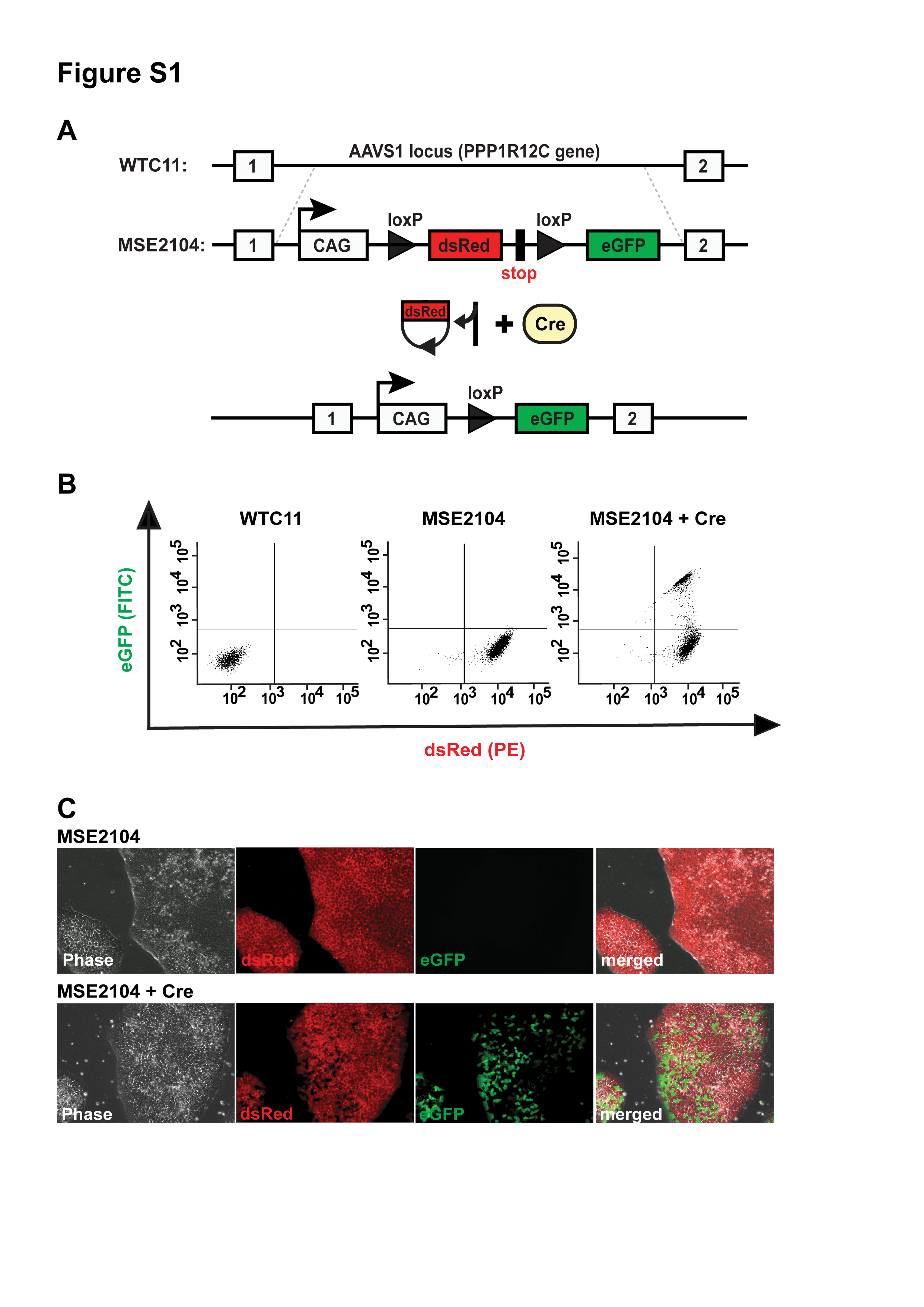

Supplement: Figure S1 — MSE2104 iPSC line with a Cre-dependent dual-fluorescent reporter cassette. [file jvi.01595-23-s0001.tif]

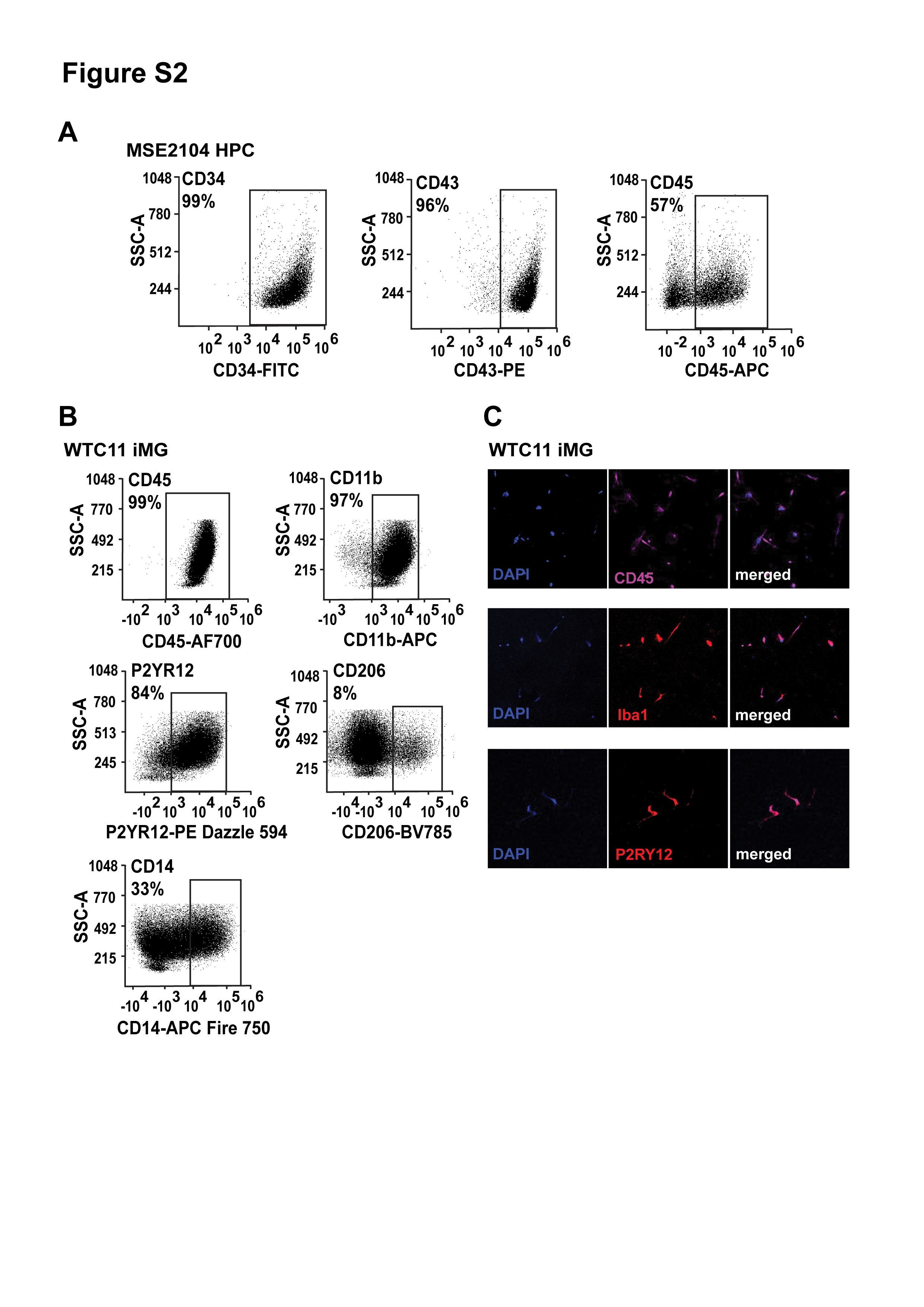

Supplement: Figure S2 — MSE2104 iPSC differentiate into hematopoietic progenitor cells (HPC) and microglia (iMG) in vitro. [file jvi.01595-23-s0002.tif]

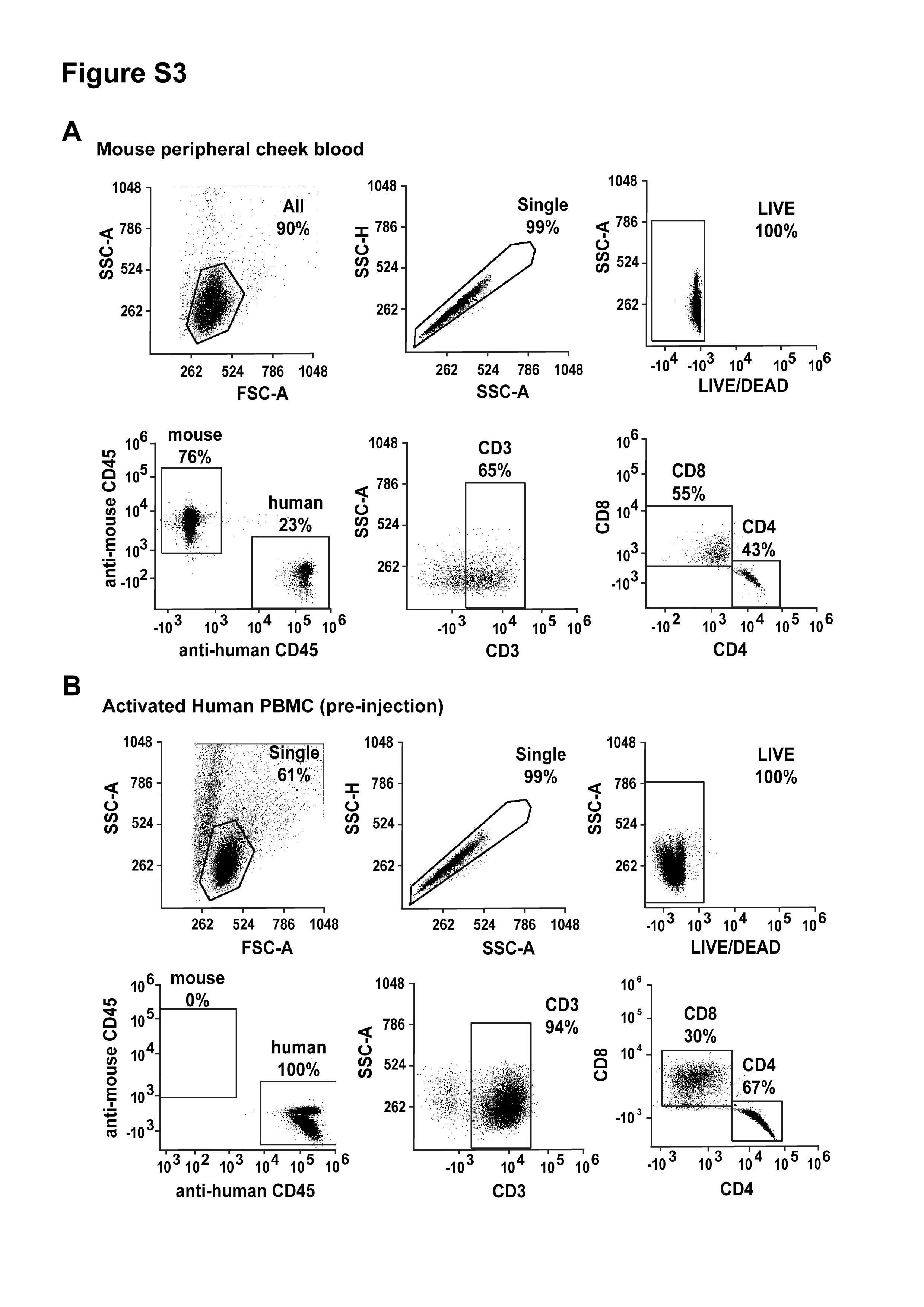

Supplement: Figure S3 — Quality assessment of peripheral blood T-cells. [file jvi.01595-23-s0003.tif]

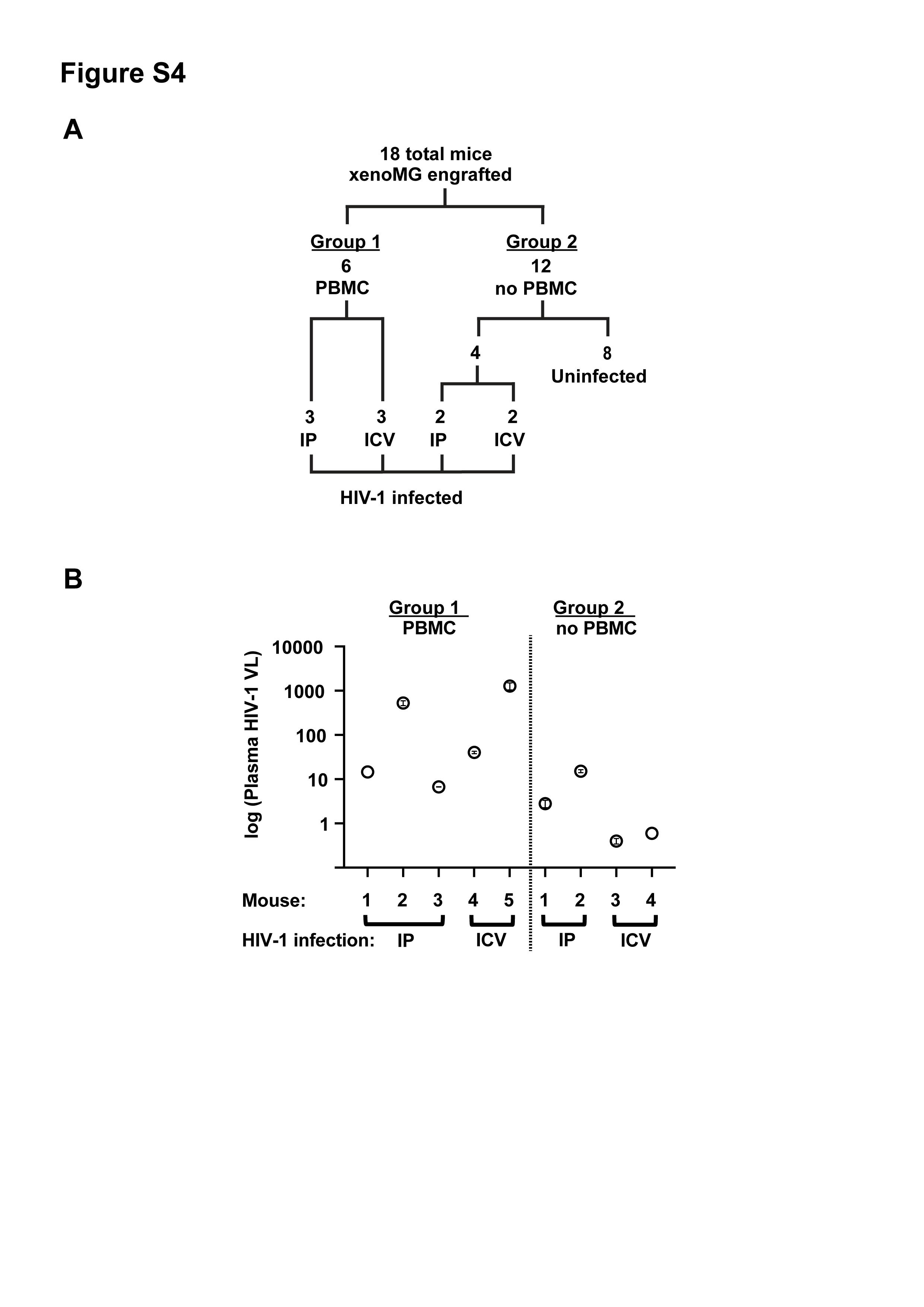

Supplement: Figure S4 — Two modes of HIV-1 infection tested in xenoMG mice with and without peripheral huPBMC. [file jvi.01595-23-s0004.tif]

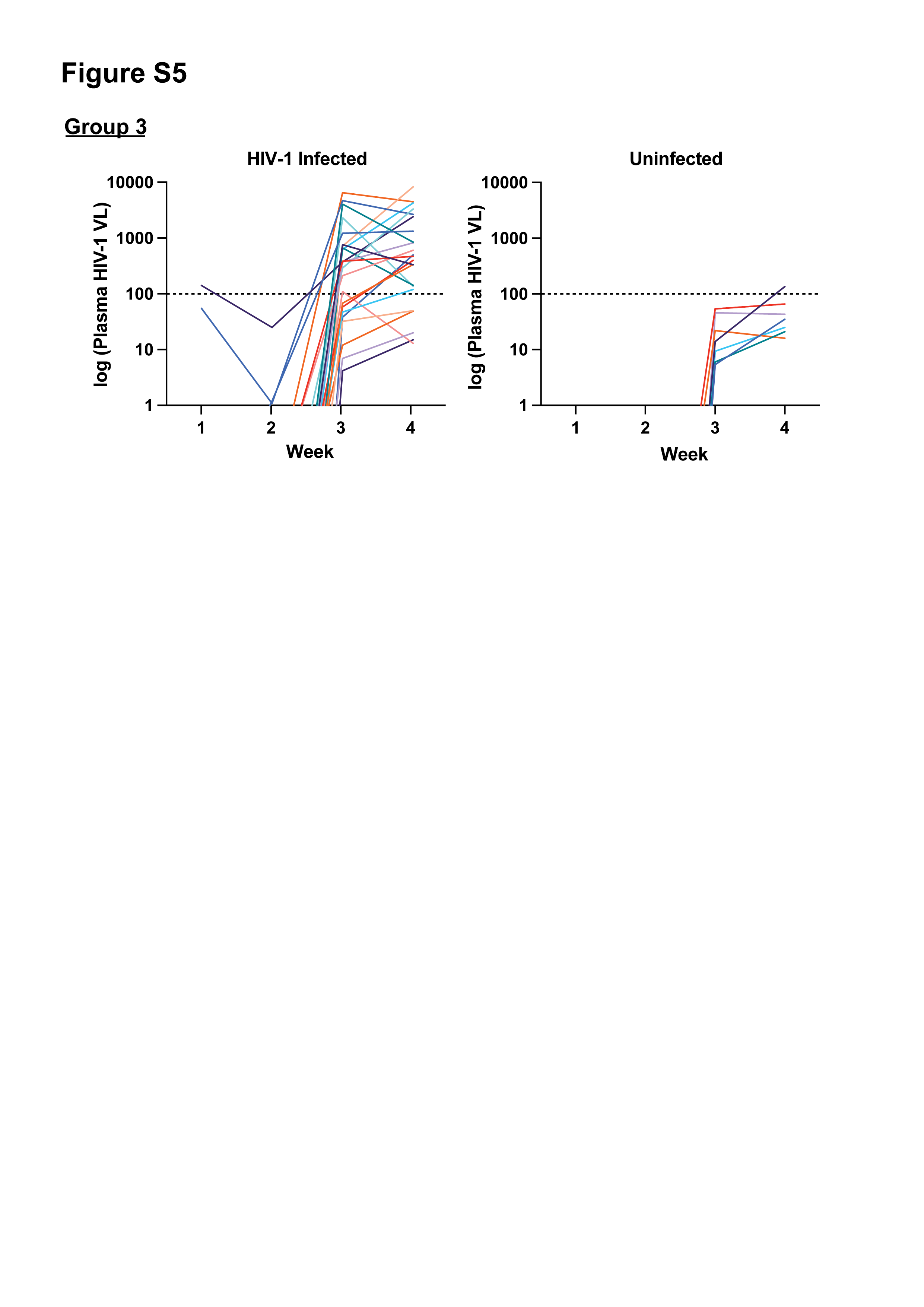

Supplement: Figure S5 — Time-course plasma HIV-1 viral load in dually xenografted humanized mice. [file jvi.01595-23-s0005.tif]
