# Supplementary material for: HIV-1 infection of genetically engineered iPSC-derived central nervous system-engrafted microglia in a humanized mouse model
Source: J Virol. 2023 Nov 30;97(12):e01595-23. doi: 10.1128/jvi.01595-23 (PMC10734545; doi:10.1128/jvi.01595-23)
Supplement: Supplemental legends — Legends for Figures S1 to S5 and Table S1. [file jvi.01595-23-s0006.docx]

**Supplementary Figure Legends**

**Figure S1**. **MSE2104 iPSC line with a Cre-dependent dual-fluorescent reporter cassette**. (**A**) An overview of the Cre-sensitive reporter system developed from the parental WTC11 iPSC line that was genetically modified at the AAVS1 locus; the reporter system was validated (**B**) by FACS analysis and (**C**) by phase contrast microscopy, wherein the iPSCs were only dsRed+ at baseline. Some cells switched from dsRed to eGFP after transfection with a Cre-expressing plasmid.

**Figure S2**: **MSE2104 iPSC differentiate into hematopoietic progenitor cells (HPC) and microglia (iMG**) ***in vitro*.** The differentiated cells were validated using flow cytometry analysis, where: (**A**) iPSC-derived HPCs expressed CD34, CD43, and CD45 cell surface markers, and (**B**) iPSC-HPC-derived microglia (iMG) expressed CD45, CD11b, P2YR12 cell surface markers. Cells were also stained with CD206 and CD14 to gate out other myeloid lineage cells. (**C**) Immunocytochemical staining of iMGs also confirmed the expression of CD45 and myeloid/microglia-specific markers Iba1 and P2RY12.

**Figure S3**. **Quality assessment of peripheral blood T-cells**. **(A)** Mouse peripheral cheek blood was collected four weeks after injection with huPMBC to check for human immune cell engraftment. A sizeable portion of CD4^+^ and CD8^+^ T-cell fraction was observed. **(B)** Representative human immune cell surface marker expression profile of CD3-gated CD4^+^ and CD8^+^ T-cell population in an activated (pre-injection) huPBMC sample.

**Figure S4. Two modes of HIV-1 infection tested in xenoMG mice with and without peripheral huPBMC**. (**A**) Diagram representing the experimental mice (total n=18) used in this experiment. Group 1 mice were dually engrafted, centrally with xenoMG and peripherally with huPBMC. Group 2 mice were singly engrafted, only centrally with xenoMG. Two groups were randomized to two routes of HIV-1 infection – IP and ICV – in those mice selected for HIV infection. Some Group 2 mice were reserved as uninfected control mice for comparison. (**B**) Peripheral blood HIV-1 viremia was measured in Group 1 and Group 2 mice, and those values are plotted as a log function in this graph. One ICV HIV-1 infected mouse in Group 1 died prematurely; hence, we could not measure HIV-1 viral load. Errors bars represent the SEM of three technical replicates.

**Figure S5. Time-course plasma HIV-1 viral load in dually xenografted humanized mice.** Weekly cheek blood plasma HIV-1 viral load was measured in a cohort (Group 3) of (Left) HIV-1 infected (n=23) and (Right) uninfected (n=7) mice. Each curve represents a single mouse whose plasma viremia was trended over the course of 4 weeks. See **Table S1** for raw data. Dotted line represent threshold above background signal.

**Table S1.** Weekly cheek blood plasma HIV-1 viral load in HIV-1 infected mice. The asterisk (*) refers to HIV-1 copy/mL <100 that is below the threshold level of detection.
